# Identifying Drug Targets of Oral Squamous Cell Carcinoma through a Systems Biology Method and Genome-Wide Microarray Data for Drug Discovery by Deep Learning and Drug Design Specifications

**DOI:** 10.3390/ijms231810409

**Published:** 2022-09-08

**Authors:** Yi-Chung Lin, Bor-Sen Chen

**Affiliations:** Laboratory of Automatic Control, Signaling Processing and Systems Biology, Department of Electrical Engineering, National Tsing Hua University, Hsinchu 30013, Taiwan

**Keywords:** oral squamous cell carcinoma (OSCC), deep neural network-based drug–target interaction (DNN-based DTI) model, genome-wide genetic and epigenetic network (GWGEN), significant biomarkers, drug design specifications

## Abstract

In this study, we provide a systems biology method to investigate the carcinogenic mechanism of oral squamous cell carcinoma (OSCC) in order to identify some important biomarkers as drug targets. Further, a systematic drug discovery method with a deep neural network (DNN)-based drug–target interaction (DTI) model and drug design specifications is proposed to design a potential multiple-molecule drug for the medical treatment of OSCC before clinical trials. First, we use big database mining to construct the candidate genome-wide genetic and epigenetic network (GWGEN) including a protein–protein interaction network (PPIN) and a gene regulatory network (GRN) for OSCC and non-OSCC. In the next step, real GWGENs are identified for OSCC and non-OSCC by system identification and system order detection methods based on the OSCC and non-OSCC microarray data, respectively. Then, the principal network projection (PNP) method was used to extract core GWGENs of OSCC and non-OSCC from real GWGENs of OSCC and non-OSCC, respectively. Afterward, core signaling pathways were constructed through the annotation of KEGG pathways, and then the carcinogenic mechanism of OSCC was investigated by comparing the core signal pathways and their downstream abnormal cellular functions of OSCC and non-OSCC. Consequently, HES1, *TCF*, NF-κB and SP1 are identified as significant biomarkers of OSCC. In order to discover multiple molecular drugs for these significant biomarkers (drug targets) of the carcinogenic mechanism of OSCC, we trained a DNN-based drug–target interaction (DTI) model by DTI databases to predict candidate drugs for these significant biomarkers. Finally, drug design specifications such as adequate drug regulation ability, low toxicity and high sensitivity are employed to filter out the appropriate molecular drugs metformin, gefitinib and gallic-acid to combine as a potential multiple-molecule drug for the therapeutic treatment of OSCC.

## 1. Introduction

In recent years, oral cancer has been the eighth most common malignancy of the head and neck, with more than 145,500 deaths worldwide each year [1,2], and oral squamous cell carcinoma (OSCC) accounts for approximate 90% of all cancers in the oral cavity [3]. Despite many advancements in cancer treatment, the 5-year survival rate for OSCC patients is 50% [4], which has remained unchanged over the past decade. In recent years, various environmental factors, such as smoking and chewing betel nut, are the main causes of OSCC [5]. However, not everyone exposed to these triggers will develop oral cancer. Many studies have shown that the occurrence of oral cancer is the result of oncogene activation or tumor suppressor gene inactivation. Therefore, a better understanding of the regulatory networks between molecular interactions and signaling pathways is crucial for identifying new prognostic markers or therapeutic targets for OSCC.

Chronic inflammation can promote tumor formation. The long-term chronic inflammatory stimulation of periodontal tissue can form an inflammatory microenvironment that is conducive to the development of OSCC [6]. Inflammation activates NF-κB through intracellular signaling pathways, induces the cytokines tumor necrosis factor-α (TNF-α) expression of cytokines and can form an inflammatory microenvironment that promotes tumor growth and progression [7]. Long-term inflammatory damage induces cell renewal and the repair of defective tissues. During the repair process, carcinogens or macrophages can cause cell DNA damage, cell proliferation and differentiation. Disruption occurs, creating conditions for the formation and metastasis of OSCC [8]. NF-κB can also upregulate vascular endothelial growth factor (VEGF) and cyclooxygenase-2 (COX-2) expression [9,10], inducing tumor angiogenesis involved in the invasion and metastasis of OSCC.

**Figure 1:**
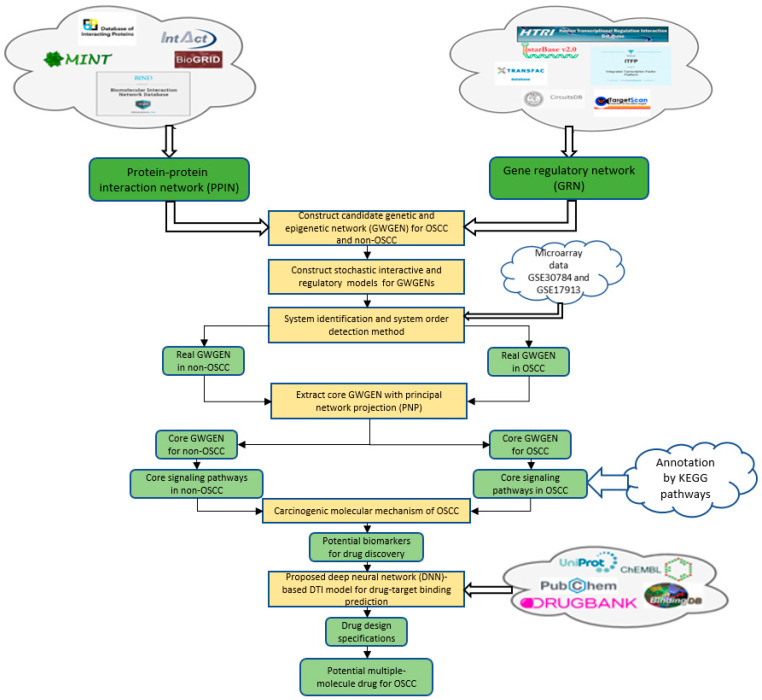
Flowchart of the systems biology method and the outline of the systematic drug discovery design. The candidate GWGEN consists of a gene regulation network (GRN) and protein–protein interaction network (PPIN), where the candidate GRN was constructed through integrating gene regulation databases, and candidate PPI was constructed via protein–protein interaction databases. The candidate GWGEN was identified to obtain real GWGEN by OSCC microarray data from GSE30784 and GSE17913 through system identification and system order detection, and core GWGEN was extracted from real GWGEN by the PNP method. The core signaling pathways of non-OSCC and OSCC are obtained by core GWGENs of non-OSCC and OSCC via the denotation of KEGG pathways, respectively. The carcinogenic biomarkers were identified by comparing the core signaling pathways and their down streaming abnormal cellular functions of non-OSCC and OSCC. The DNN-based DTI model can be employed to predict candidate molecular drugs for these carcinogenic biomarkers, and drug design specifications are used to select a multiple-molecule drug for OSCC.

In this study, based on big data mining, the system identification method and genome-wide microarray data of patients of OSCC, a systems biology method is proposed to help us analyze macroscopically systematic relationships among proteins, genes and microenvironments in cancer. As shown in Figure 1, the systems biology method including system identification, system order detection and principle network projection (PNP) [11,12] has been widely used to construct core signal pathways to investigate the pathogenesis of diseases such as cancer [13] and virus infection [14] in recent years. Therefore, based on genome-wide microarray data of OSCC and non-OSCC, a systems biology method is used to find the carcinogenic mechanism of OSCC by comparing core signal pathways and their downstream abnormal cellular functions of OSCC and non-OSCC in this study. First, a systems biology method was employed to identify real GWGENs of OSCC and non-OSCC by prune false positives from the candidate GWGEN by their microarray data. Then, with the PNP approach, the core GWGENs of OSCC and non-OSCC were extracted from their real GWGENs, respectively. By the annotation of KEGG pathways, we could obtain core signal pathways of OSCC and non-OSCC from their corresponding core GWGENs. Then, we can investigate the carcinogenic mechanism of OSCC by comparing the discrepancy between core signal pathways and their downstream cellular disfunctions in the non-OSCC and OSCC. According to the investigated carcinogenic mechanism and cellular disfunctions in the core signaling pathways of OSCC, HES1, *TCF*, NF-κB and SP1 were identified as the significant carcinogenic biomarkers contributing to unnormal cellular functions such as inflammatory-dependent cell apoptosis, angiogenesis, tumor metastasis and tumor invasion, which were considered as drug targets for the systematic drug discovery design of OSCC.

The development process of a new drug is an arduous task because of the highly expensive cost and time-consuming investment. Moreover, it is estimated that it takes about 12–15 years and more than USD one billion to develop a new drug [15]. Pharmaceutical companies need to spend a large amount of time and effort on executing experiments to understand the properties and the possible bindings of the drug and selected targets. In addition, the efficacy and potency of the drug as well as the adverse influences on body should be considered. Therefore, researchers conduct a number of animal and clinical trials to check the safety and stability [16]. These complicated procedures vastly increase the risk of failure in designing drugs. Most failures are due to the poor clinical outcomes, and the results are usually lower than expected [17]. On the contrary, drug repurposing (also known as drug repositioning) has been employed to identify new therapeutic uses of approved or investigational drugs as a feasible and advantageous strategy [18]. Recently, deep learning schemes have been widely applied for some phenomenon predictions of molecular biological systems [19,20,21]. For these reasons, we developed systematic drug discovery and design strategies based on a DNN-based DTI model to predict candidate molecular drugs for biomarkers (drug targets) of OSCC. Then, these candidate molecular drugs of each drug target were sieved by drug design specifications of drug–target interaction (docking), adequate drug regulation ability, low toxicity and high sensitivity to select adequate molecular drugs for biomarkers, which are combined as a multiple-molecular drug of OSCC, from the perspective of system engineering. As seen in the flowchart of the systematic drug design procedure in a DNN-based DTI model was trained by DTI databases in advance. Then, the well-trained DNN-based DTI model could predict a set of candidate molecular drugs for each biomarker (drug target). Eventually, with the help of the above drug design specifications, we chose and combined metformin, gefitinib and gallic-acid among the set of candidate molecular drugs as the multiple-molecule drug to target the biomarkers HES1, *TCF*, NF-κB and SP1 for OSCC. Taken together, we expect that the proposed systematic medicine discovery and design procedure can provide an efficient way to design a multiple-molecule drug as a new therapy for OSCC treatment before clinical trials.

## 2. Results

### 2.1. Overview of the Systems Biology Method and the Systematic Drug Discovery and Design of OSCC

**Figure 2:**
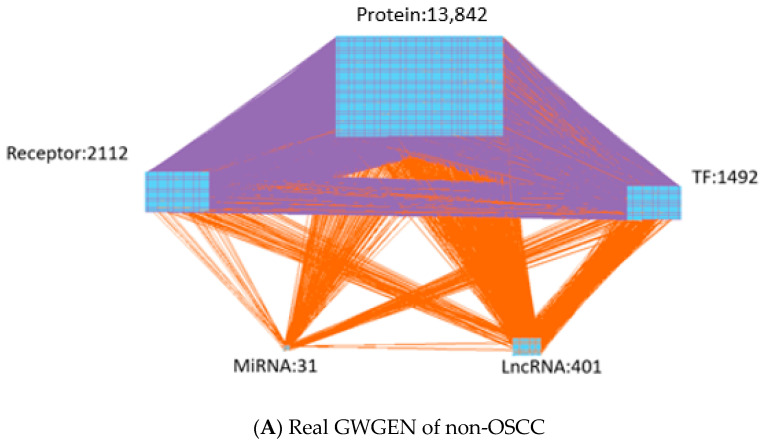

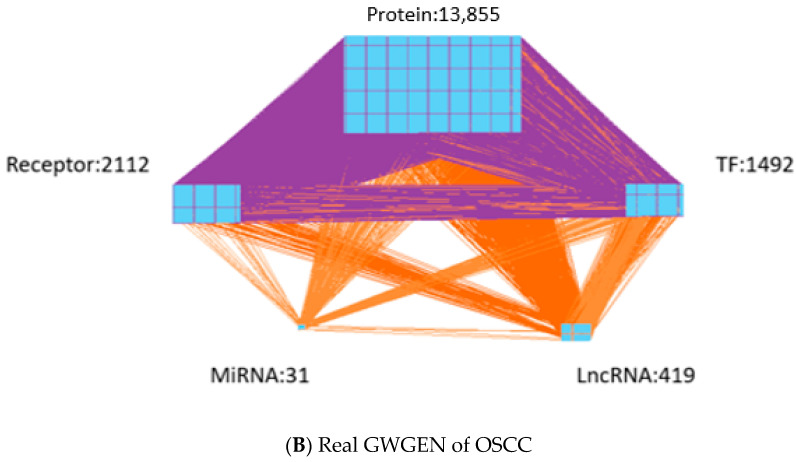
(**A**) The real GWGEN of non-OSCC. (**B**) The real GWGEN of OSCC. The numbers indicate the node numbers of proteins, TFs, Receptors, LncRNAs and miRNAs. The purple lines indicate the protein–protein interactions, and the orange lines denote the gene regulations.

**Figure 3:**
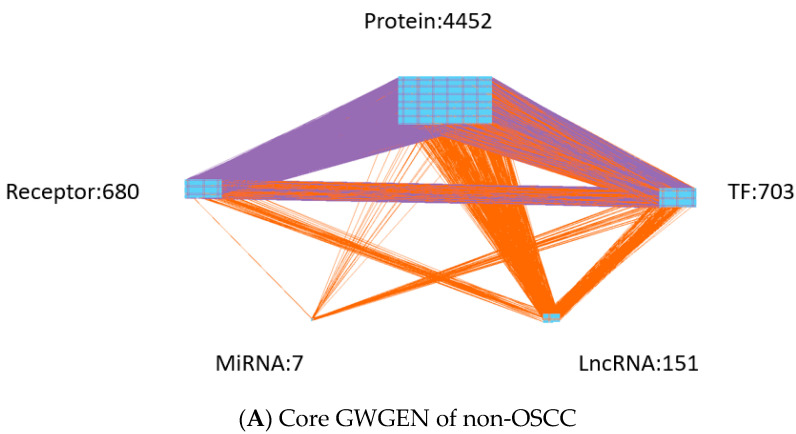

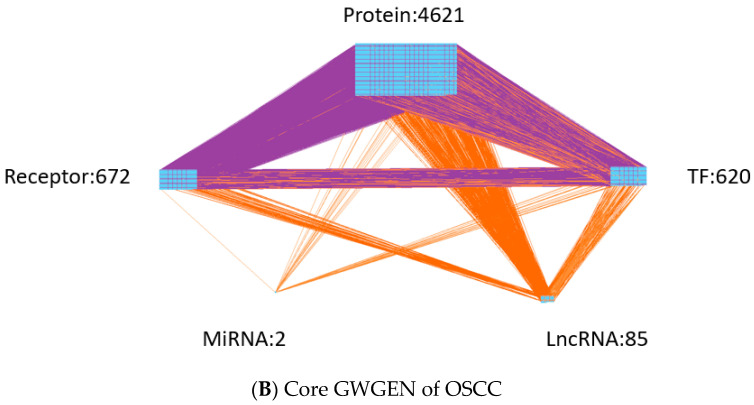
(**A**) The core GWGEN of non-OSCC. (**B**) The core GWGEN of OSCC. The core GWGENs were extracted by the PNP method from the real GWGENs to simplify the annotation of KEGG pathways for the carcinogenic mechanism analysis of OSCC. The numbers denote the node numbers of proteins, TFs, Receptors, LncRNAs and miRNAs, respectively. The purple lines indicate the protein–protein interactions, and the orange lines denote the gene regulations.

**Figure 4:**
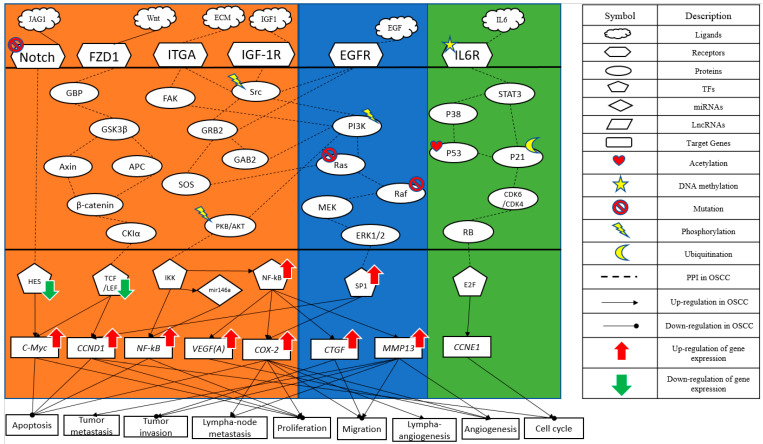
The core signaling pathways in three blocks represent specific OSCC, common non-OSCC and specific non-OSCC core signaling pathways from left to right, respectively. The core signaling pathways of non-OSCC and OSCC are based on the annotation of core GWGENs of non-OSCC and OSCC in Figure 3, respectively. For investigating the genetic and epigenetic carcinogenic mechanism of OSCC, the core signaling pathways and the downstream abnormal cellular functions of non-OSCC and OSCC are compared. The genes and proteins in the core signaling pathways were chosen from core GWGENs of the non-OSCC and OSCC by the annotation of KEGG pathways. The gene regulations and protein interactions were constructed based on the edges in core GWGENs of non-OSCC and OSCC. The low and high expression arrow-head marks are relative to non-OSCC.

**Figure 5:**
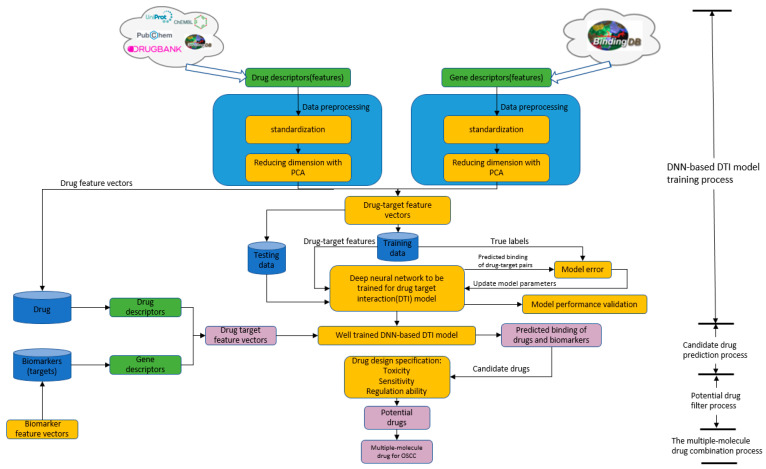
The flowchart of the systematic drug discovery and design procedure for OSCC. The drug–target binding datasets were obtained from the binding database BindingDB, which integrates the information of drugs and targets from several databases. Then, the drug and target features were preprocessed respectively, including descriptor transformation, standardization and PCA dimension reduction. Afterwards, the processed data were split into the training data for DNN-based DTI model training and the testing data for DTI model performance validation in Figure 5 and Figure 6. We updated the model parameters through the model error between the true binding label and the predicted binding label of drug–target pairs. The well-trained DNN-based DTI model was used to predict the binding probability between drugs and targets (biomarkers). Therefore, candidate drugs were predicted for each biomarker in Table 5 by the well-trained DNN-based DTI model from the drug databases and further filtered as potential drugs by the drug design specifications of suitable regulation ability, low toxicity and high sensitivity, which are combined as a multiple-molecule drug for OSCC in Table 6.

In order to obtain insight into the carcinogenic mechanism to identify significant carcinogenic biomarkers as drug targets of OSCC, we search for potential molecular drugs to target these significant biomarkers by a deep neural network (DNN)-based DTI model and drug design specifications from the viewpoint of system engineering. The first step is to construct a candidate GWGEN of non-OSCC and OSCC by big data mining from the databases DIP [22], IntAct [23], BioGRID [24], MINT [25] HTRIdb [26], ITFP [27], TRANSFAC [28], CircuitDB [29], TargetScanHuman [30] and starBase 2.0 [31]. Then, the system identification method in Equations (1)–(24) by the microarray data of non-OSCC and OSCC and the system order detection method in Equations (25)–(32) are employed to construct real GWGENs of non-OSCC and OSCC in Figure 2 by pruning off the false positives from candidate GWGEN, respectively. Since, at most, 6000 molecules in real GWGENs can be annotated by the Kyoto Encyclopedia of Genes and Genomes (KEGG) pathways, the principal network projection method (PNP) in Equations (33)–(40) is used for extracting the core GWGENs in Figure 3, i.e., the core GWGEN network of non-OSCC and the core GWGEN network of OSCC are at the maximum with 6000 significant nodes. The core GWGENs extracted by the PNP method from the real GWGENs can also simplify the investigation of the carcinogenic mechanism of OSCC. The numbers of proteins, TFs, Receptors, LncRNAs and miRNAs of core GWGENs are also indicated. The core signaling pathways of non-OSCC and OSCC are constructed by projecting the corresponding core GWGENs to KEGG significant pathways in Figure 4. By comparing the core signaling pathways and the downstream abnormal cellular functions between non-OSCC and OSCC in Figure 4, we could investigate the carcinogenic mechanism of OSCC, from which the significant biomarkers were identified as drug targets for the therapeutic treatment of OSCC. Furthermore, the deep neural network of the drug–target interaction (DTI) model is trained in Figure 5 to predict candidate molecular drugs, which are selected by drug design specifications such as adequate drug regulatory ability, low toxicity and high sensitivity as potential molecular drugs that can be combined as a multiple-molecule drug of OSCC.

The collected microarray data were classified into non-OSCC and OSCC groups, as shown in Table 1.

Based on protein interaction and gene regulation databases, since the candidate GWGEN was constructed due to different biological conditions and computational predictions in these databases, there are many false positives in the candidate GWGEN. Therefore, the system identification method in Systems Biology [32,33] is employed to identify the protein interactions and gene regulations by the microarray data of OSCC and non-OSCC. Systems order detection is employed to prune off the false positive protein interactions out of the interaction order of each protein and the gene regulations out of the regulation order of each gene in candidate GWGEN to obtain the real GWGENs of OSCC and non-OSCC. The real GWGENs were too complex and large for analyzing the carcinogenesis of OSCC by the annotation of KEGG pathways. To solve this problem, the core GWGENs were extracted from the real GWGENs of OSCC and non-OSCC by the PNP method to reduce the network size in order to simplify the following carcinogenic analysis of OSCC by the annotation through KEGG pathways. In Table 2, the sizes of the core GWGENs of OSCC are smaller than real OSCC GWGENs. Both the real OSCC GWGEN and the real non-OSCC were plotted by Cytoscape software in Figure 2. The corresponding core GWGENs for non-OSCC and OSCC were plotted by Cytoscape software in Figure 3. Moreover, the core signaling pathways were obtained by the annotation of core GWGENs by KEGG pathways to investigate the carcinogenic mechanism by comparing core signaling pathways. The KEGG pathway enrichment analyses of the core signaling pathways of OSCC and non-OSCC are given in Table 3 and Table 4, respectively. The core signaling pathways for OSCC and non-OSCC are given in Figure 4. Then, based on the core signal pathways and their downstream abnormal cellular functions of OSCC and non-OSCC in Figure 4, we will investigate the genetic and epigenetic carcinogenic mechanism of OSCC in the following.

The content in the table shows the number of nodes. The rows of the table contain different types of nodes.

### 2.2. Investigating the Genetic and Epigenetic Carcinogenic Mechanism of OSCC

Cancer is a disease mainly caused by abnormal signaling pathways. Smoking and drinking can cause changes in the microenvironment of the human body [34,35]. In our study, adverse factors such as JAG1, Wnt, ECM, EGF and IGF1 produced by the human microenvironment ultimately lead to cellular dysfunctions. From comparing the core signaling pathways of OSCC and non-OSCC in Figure 4, the core signaling pathways of OSCC and their downstream abnormal cellular functions need to be investigated into the genetic and epigenetic carcinogenic mechanism of OSCC. The core signal pathways and their downstream cellular functions of OSCC are investigated as follows:

(i) Abnormal MAPK signaling pathway in OSCC

In Figure 4, the ligand epithelial growth factor (EGF) in the micro-environment of OSCC targets the EGF receptor (EGFR), activates the phosphorylation of its downstream signaling proteins Src and PI3K and then leads to the well-known estrogen-mediated Ras/Raf/MEK/ERK pathway [36]. Several mutations in the MAPK/ERK pathway have been identified in human cancers. The mitogen-activated protein kinase (MAPK) cascade is a critical pathway for human cancer cell survival, dissemination and resistance to drug therapy. The extracellular signal-regulated kinase (ERK) pathway is a convergent signaling node that receives input from numerous stimuli, including internal metabolic stress and DNA damage pathways and altered protein concentrations, as well as from external growth factors, cell–matrix interactions and communications from other cells. Mutated genes responsible for regulating cell fate, genome integrity and survival can overactivate this pathway by causing increased protein amplification and altering the tumor microenvironment. These mutations can occur upstream of membrane receptor genes [37]. Current and future drug development efforts will require altering and modulating tumor signaling in this complex network of codependent pathways. ERK ultimately redirects to the transcription factor SP1, which is abnormally upregulated in patients with OSCC, and SP1 promotes the expression of CCND1 and COX-2 [38,39]. COX-2 has been reported to be involved in cancer cell migration, cancer cell proliferation, lymph-angiogenesis and metastasis. There was a significant positive correlation between angiogenesis and apoptosis [40]. CCND1 was negatively correlated with apoptosis and an accelerated cell cycle [41,42].

(ii) The impact of the Wnt signaling pathway on OSCC

The ligand Wnt is common in embryonic development and cancer [43]. Wnts are secreted glycoproteins that bind to frizzled class 1 (FZD1) receptors, which may be coupled to heterotrimeric G proteins. Intracellularly, signal transduction passes through GBP, glycogen synthase kinase 3β (GSK3β) and tumor suppressor gene product (APC) and then activates a key protein (β-catenin). Consequentially, stable β-catenin enters the nucleus and interacts with TF *TCF/LEF*, leading to the transcription of the Wnt target genes *C-Myc* and CCND1 [44]. According to reports and studies [45], there is a regulation of underexpressed TF *TCF/LEF* on *C-Myc* and CCND1. CCND1 was positively correlated with apoptosis and an accelerated cell cycle. *C-Myc* was positively correlated with proliferation and negatively correlated with apoptosis [46].

(iii) Notch signaling pathway in OSCC

In Figure 4, the cell surface receptor Notch is activated and causes mutation by the microenvironmental factor JAG1 of OSCC [47,48]. The constitutive activation of the Notch pathway has been demonstrated in various types of malignancies [49]. In this study, the Notch pathway was also found to be extremely important in OSCC. The Notch signaling cascade affects several key aspects of normal development through proliferation and apoptosis [50]. All the signaling pathways transduce down to the TF HES1 [51]. Finally, the signal is transmitted to the proto-oncogene *C-Myc* [52]. According to reports and our study, HES1 is underexpressed and upregulates *C-Myc* and then promotes cell apoptosis and proliferation in OSCC patients [50].

(iv) Key transcription factor NF-κB on OSCC

Integrin subunit alpha (ITGA) also plays a crucial role in OSCC. In Figure 4, it can be seen that, after the receptor ITGA is affected by the extrinsic factor extra cellular matrix (ECM) in the micro-environment of OSCC [53], it will then successively affect the downstream signaling transduction proteins Src/PI3K/PKB/AKT. Insulin-like growth factor 1 (IGF-1R) signaling is partially mediated by the Src pathway. The activation of Src and IGF-1R also activates Akt. It is a key effector of the PI3K/Akt pathway. It is abnormally activated in most malignant tumors, promoting cell growth, proliferation and survival [54]. In many reports, AKT is underexpressed and phosphorylated in OSCC patients [55]. It then affects the TFs IKK and NF-κB. NF-κB affects numerous target genes. Many reports indicate that the genes COX-2 and VEGF are particularly important in OSCC patients [55,56]. The genes NF-κB, COX-2 and VEGF are abnormally up-regulated to promote angiogenesis, lympha-angiogenesis, migration, proliferation and apoptosis [55,56].

According to the description of OSCC above, poor diet and living habits can lead to changes in the human microenvironment. Then, a series of pathway signaling leads to downstream cellular dysfunction, among which abnormal apoptosis, proliferation, migration and angiogenesis play crucial roles on the carcinogenesis of OSCC.

### 2.3. Significant Biomarkers as Drug Targets for the Therapeutic Treatment of OSCC Utilizing the Systematic Drug Discovery Approach

After investigating the carcinogenic mechanism of OSCC from the core signaling pathways and their downstream abnormal cellular functions, the significant biomarkers of the carcinogenic mechanism of OSCC will be identified as drug targets for therapeutic treatment.

According to the investigation of carcinogenic mechanisms, OSCC suffers from proliferation and apoptosis. Based on the above core signaling pathways and their downstreaming abnormal cellular functions, we properly selected significant biomarkers that were related to carcinogenically abnormal inflammation, apoptosis, proliferation, angiogenesis and cell cycles. Consequently, we selected HES, *TCF*, NF-κB and SP1 as significant biomarkers and aimed to reverse their expression levels, i.e., to restore them to normal inflammation, apoptosis, proliferation, angiogenesis and cell cycles. HES plays an important role in the NOTCH pathway. *TCF* is also a main character in the Wnt pathway. NF-κB is involved in cellular responses to many stimuli. Sp1 has been shown to be involved in apoptosis.

After identifying the significant biomarkers as drug targets, we considered the chemical properties of drugs and targets to select candidate molecular drugs for these drug targets (biomarkers) based on their drug–target bindings (dockings) predicted by the DNN-based DTI model and to design a multiple-molecule drug for OSCC treatment before clinical trials based on some suitable drug design specifications, i.e., adequate drug regulation ability, low toxicity and high sensitivity. The flowchart of the systematic drug discovery and design method is described in Figure 5.

**Figure 6:**
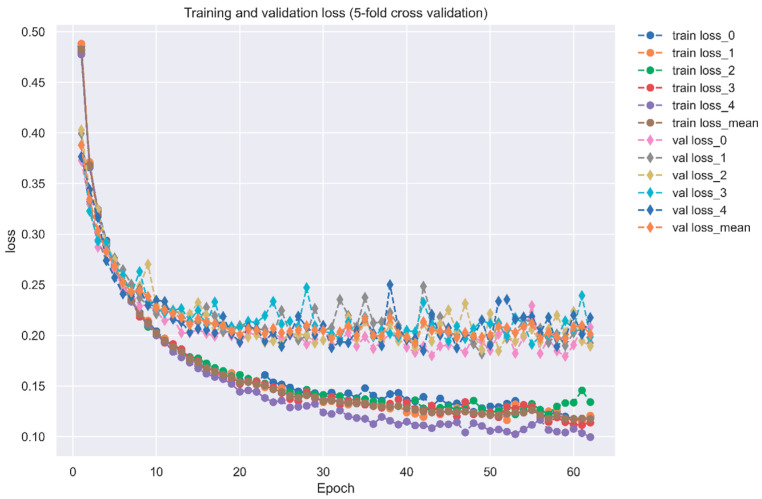
Training and validation loss of the DNN-based DTI model (five-fold cross validation).

**Figure 7:**
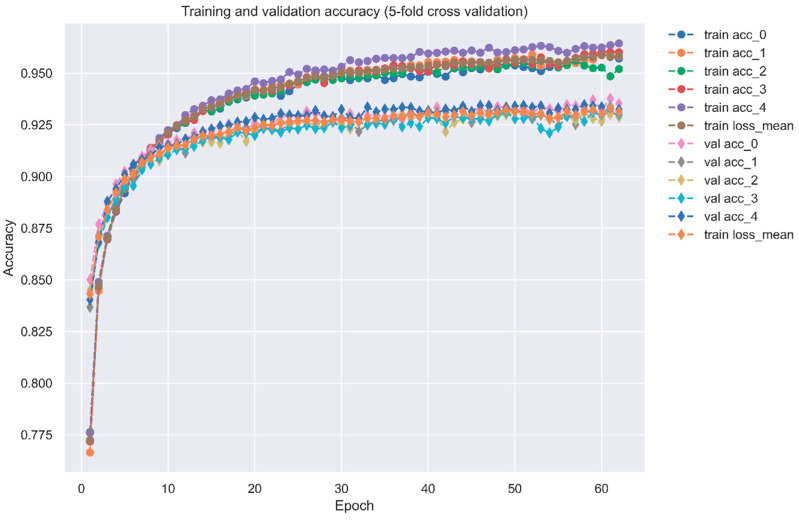
Training and validation accuracy of the DNN-based DTI model (five-fold cross validation).

For our DNN-based DTI model in Figure 5, DNN was set with four hidden layers, and each of them is connected with a ReLU activation function layer after it. ReLU activation function could avoid vanishing gradient problems and converge much faster than the other activation functions. Although ReLU is not the best activation function, it is useful for the classification issue. Additionally, the dropout layer is incorporated after each hidden layer to prevent overfitting. The input layer has 996 nodes, and 512, 256, 128 and 64 neurons are embedded, respectively, in four hidden layers. Before the output layer, a sigmoid activation function is used to restrict the output value in the range of 0 to 1 as a probability value. In general, the sigmoid function is usually used for binary classification. The outcome of DTI is a probability value, where a higher value corresponds to a more reliable interaction (docking) between the drug and the target. The loss and accuracy during the training process are separately recorded in Figure 6 and Figure 7, respectively.

**Figure 8:**
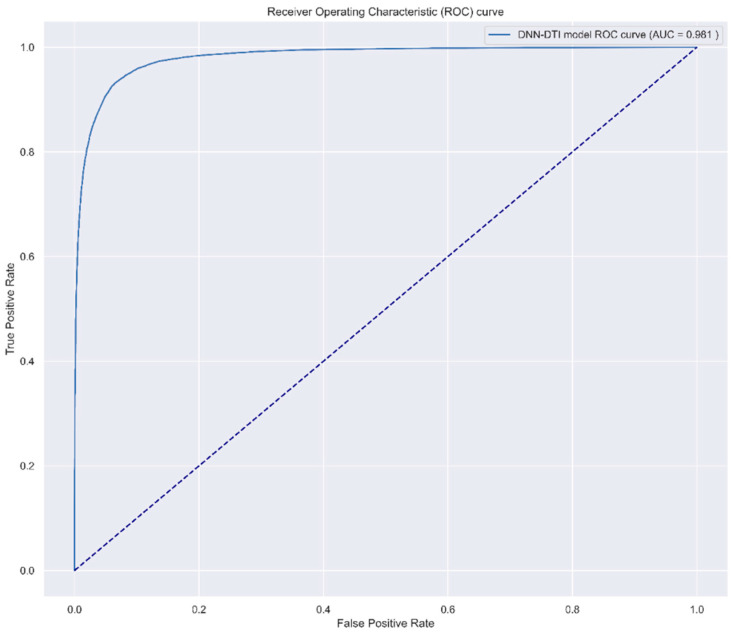
The receiver operating characteristic (ROC) curve measure of the probability of the prediction accuracy of the DNN-based DTI model, with the area under the curve of ROC (AUC-ROC) score of 0.981.

Furthermore, we also plot the receiver operating characteristic (ROC) curve measure of the probability of the prediction accuracy of the DNN-based DTI model. The visualization of the ROC curve comparison is denoted in Figure 8. From Figure 8, the prediction performance of our proposed DTI model indicates that the deep learning concept is promising to adapt to the large and complicated drug–target interaction data.

To find suitable drug candidates, predictions are made based on the high probability of the candidate drug binding (docking) to the selected biomarker. At the same time, since powerful drugs are often associated with a high risk of damage, attention should also be paid to the balance between drug efficacy and adverse effects. Correspondingly, we guarantee the stability and safety of the drug in clinical trials, taking into account the drug design specifications, such as regulatory ability, toxicity and sensitivity.

To measure the regulatory ability of drug candidates, available regulatory capacity data were downloaded from the L1000 level5 dataset [57,58], which contains 978 genes treated with 19,811 small molecule compounds in 78 different cell lines. In the accommodation ability data, positive values indicate up-regulations and negative values indicate down-regulations. Based on this criterion, we searched for molecular drugs in suitable cell lines that could reverse the expression of carcinogenic biomarkers in OSCC to restore their normal expressions to remedy their down streaming cellular dysfunctions of OSCC. In addition, a lower drug toxicity has the effect of reducing side effect by referring to the median lethal dose (LD50) value in DrugBank [59]. LD50 is often considered for disease and cancer drug design. The lower the LD50 value, the greater the toxicity. In addition, a higher drug sensitivity (lower EC50 value) can reduce the drug dosage. Drug susceptibility data were obtained from the PRISM dataset [60], which includes 4518 drugs tested in 578 human cell lines based on the half-maximal effective concentration (EC50). EC50 is a measure of the potency of a drug, where a lower EC50 indicates that the drug works best at lower doses. As mentioned above, aberrant expression in a disease can be well reversed by choosing the right drug. Some candidate drugs predicted by the DNN-based DTI model for identified biomarkers and their regulability, toxicity, and sensitivity information are shown in Table 5. The potential drugs metformin, gallic-acid and gefitinib are selected by the three above-mentioned drug design specifications (i.e., suitable regulation ability, high sensitivity and low toxicity) and are combined as a multiple-molecule drug for therapeutic treatment of OSCC in Table 6.

## 3. Discussion

### Potential Multiple-Molecule Drug for the Identified Biomarkers of OSCC

Recently, Cisplatin has been the most common drug for the treatment of OSCC [61,62], but its powerful side effects are daunting; for example, renal toxicity, nausea, vomiting and neurotoxicity [63]. In this study, we tried to find novel multiple-molecular drugs to treat OSCC. With the consideration of sensitivity, toxicity and regulation ability as drug specifications, we combined deep learning and systems biology methods to find the right molecular drugs for the identified biomarkers of OSCC. Ultimately, the molecular drugs metformin, gefitinib and gallic-acid are detected and combined as a multi-molecule drug in Table 6 for the therapeutic treatment of OSCC.

Gefitinib is a drug that acts on the tyrosine kinase domain of the epidermal growth factor receptor [64]. EGFR is overexpressed in certain human cancers, such as breast and lung cancer [65,66]. The excessive activation of EGFR by the ligand EGF in the microenvironment of OSCC leads to the abnormal activation of anti-apoptotic Ras cell signaling, resulting in uncontrolled cell division [67,68]. Gefitinib binds covalently to the enzyme adenosine triphosphate (ATP) and inhibits the epidermal growth factor receptor tyrosine kinase [69].

Metformin, a low-cost antidiabetic drug [70], has been widely used to treat diabetes by inhibiting hepatic gluconeogenesis and enhancing glucose uptake by skeletal muscle [71]. Several studies have shown that metformin is being repurposed as an anticancer therapeutic for different types of cancer [72]. The insulin receptor transmits signals through growth factor receptor binding protein 2 (GRB2) to the Ras/Raf/ERK pathway that drives cell growth. There is evidence that these pathways play an important role in the changes in cellular metabolism that are typical of tumor cells. Elevated circulating insulin/IGF1 levels and the upregulation of insulin/IGF receptor signaling have been implicated in the development of various cancers. Metformin was found to reduce insulin levels, inhibit the insulin/IGF signaling pathway and alter cellular metabolism in normal and cancer cells [73]. Interestingly, metformin can increase the sensitivity of oral cancer cells to chemotherapeutic drugs such as gefitinib [74,75], improving therapeutic efficacy and reducing dose and toxicity [76]. Overall, metformin in combination with gefitinib may be a potential drug for the development of new therapeutic strategies for human OSCC.

Gallic-acid is a phenolic compound widely found in the plant kingdom [77], such as green vegetables, fruits and other plants [78]. The toxicity of gallic-acid to normal cells of humans is very low [79]. It is highly toxic to bad cells such as fibrotic cells and cancer cells [80,81] and can kill these harmful cells; but when it encounters normal cells, the toxicity will become weaker [82].

In summary, the molecular drugs metformin, gefitinib and gallic-acid are selected and combined as a multi-molecule drug of OSCC in Table 6 from the proposed systematic drug discovery and design perspectives.

## 4. Materials and Methods

### 4.1. A General Review of Constructing Core Genome-Wide Genetic and Epigenetic Networks (GWGENs) of OSCC and Non-OSCC

At first, we divided the data into a disease group and a control group. The disease group data came from GSE30784, and the control group data came from GSE30784 and GSE17913. Then, we used the following steps to construct core GWGENs of OSCC and non-OSCC.

(1) Constructing the candidate GWGEN: We use big database mining to construct a candidate PPIN and a candidate GRN, including genes, miRNAs and lncRNAs. Note that the candidate GWGEN includes a candidate PPIN and a candidate GRN.
(2) Identifying real GWGENs: We identify the parameters of PPIN and GRN through the system identification method by solving the corresponding constrained linear least squares estimation problems with the help of the microarray data of OSCC and non-OSCC. After performing system modeling and parameter identification for proteins, genes, miRNAs and lncRNAs in the candidate GWGEN, we used the system order detection method AIC to prune the false positives in the regulation and interactions in the candidate GWGEN to obtain the real GWGENs of OSCC and non-OSCC.
(3) Extracting the core GWGENs: To extract the core GWGENs of OSCC and non-OSCC, we applied the PNP approach. By doing this, we could compute a projection value for each node in the real GWGENs to 85% significant network structures of real GWGENs. The top 6000 nodes of real GWGENs with the highest projection values have remained as core GWGENs.
(4) Building and comparing the core signaling pathways: The core signaling pathways for cells of OSCC and non-OSCC can be constructed by the annotation of the KEGG pathways of core GWGENs of OSCC and non-OSCC, respectively. We investigated the molecular mechanisms of carcinogenesis of OSCC by comparing the upstream microenvironmental factors, core signaling pathways and their corresponding downstream abnormal cellular functions of OSCC and non-OSCC.

### 4.2. Data Preprocessing for Constructing the Candidate GWGEN

In this research, we downloaded the dataset with accession numbers GSE30784 and GSE17913 from the National Center for Biotechnology Information (NCBI). We divided the data into a disease group and a control group. The disease group data came from GSE30784, and the control group data came from GSE30784 and GSE17913. Note that the candidate GWGEN included a candidate PPIN and a candidate GRN. The candidate GWGEN matrix is a binary matrix. If two nodes have an interaction or regulation, we assigned value one for it; otherwise, we assigned a value zero for it. For building the candidate PPIN, we referred to the following databases: DIP [22], IntAct [23], BioGRID [24] and MINT [25]. Moreover, to construct the candidate GRN, we considered the following databases: HTRIdb [26], ITFP [27], TRANSFAC [28], CircuitDB [29], TargetScanHuman [30] and StarBase 2.0 [31].

### 4.3. System Modeling of the Candidate GWGEN

To model the candidate GWGEN, we build system modeling for proteins, genes, miRNAs and lncRNAs. First, in the candidate GWGEN, the following interactive equation describes the *q*-th protein interaction with its *G_q_* neighboring proteins in the candidate PPIN:

$$p_{q}\left[n\right]=\sum_{\begin{array}{l} r=1 \end{array}}^{G_{q}}\kappa_{qr}p_{q}\left[n\right]p_{r}\left[n\right]+\lambda_{q,PPIM}+\mu_{q,PPIM}\left[n\right],\;\mathrm{for}q=1,\ldots ,Q,n=1,\ldots ,N$$

where $p_{q}\left[n\right]$ means the expression level of the *q*-th protein in the *n*-th sample and $p_{r}\left[n\right]$ is the expression level of the *r*-th protein in the *n*-th sample; $\kappa_{qr}$ indicates the interaction ability between the *q*-th protein and the *r*-th protein; $G_{q}$ represents the total number of proteins that interact with the *q*-th protein in the candidate PPIN; *Q* expresses the total number of proteins in the candidate PPIN; *N* denotes the total number of samples; $\lambda_{q,PPIM}$ is the basal level in the model of the *q*-th protein for unknown protein interactions of histone modifications such as phosphorylation, acetylation and ubiquitination; $\mu_{q,PPIM}\left[n\right]$ denotes the environment and measurement noise of the *q*-th protein.

Second, the transcriptional regulation of the *x*-th gene in GRN is described by the following equation:

$$\begin{array}{l} g_{x}\left[n\right]=\sum_{\begin{array}{l} u=1 \end{array}}^{U_{x}}\alpha_{xu}t_{u}\left[n\right]+\sum_{v=1}^{V_{x}}\beta_{xv}l_{v}\left[n\right]-\sum_{w=1}^{W_{x}}\gamma_{xw}m_{w}\left[n\right]g_{x}\left[n\right]+\lambda_{x,GRM}+\mu_{x,GRM}\left[n\right]\\ ,\;\mathrm{for}\;x=1,\ldots ,X,n=1,\ldots ,N \end{array}$$

where $g_{x}\left[n\right]$ means the gene expression level of the *x*-th gene in the *n*-th sample;

$t_{u}\left[n\right]$*,* $l_{v}\left[n\right]$ and $m_{w}\left[n\right]$ individually indicate the gene expression level of the *u*-th TF, the *v*-th lncRNA and the *w*-th miRNA in the *n*-th sample; $U_{x}$, $V_{x}$ and $W_{x}$ individually denote the total binding number of TFs, lncRNAs and miRNAs; $\alpha_{xu}$ is the transcriptional regulatory ability of the *u*-th TF on the *x*-th gene; $\beta_{xv}$ expresses the transcriptional regulatory ability of the *v*-th lncRNA on the *x*-th gene; $\gamma_{xw}\geq 0$ denotes the post-transcriptional regulatory ability of the *w*-th miRNA on the *x*-th gene; *X* means the total number of genes in the candidate GWGEN; *N* denotes the total number of samples; $\lambda_{x,GRM}$ means the basal level of the *x*-th gene because of the unknown gene regulations such as methylation and genetic mutation; $\mu_{x,GRM}\left[n\right]$ represents the environment or measurment noise.

Third, TFs, lncRNAs and miRNAs also have a potential impact on the *i*-th lncRNA, and we can depict this behavior by the lncRNA model (LRM) in the candidate GWGEN. The regulatory equation is described as follows:

$$\begin{array}{l} l_{i}\left[n\right]=\sum_{u=1}^{U_{i}}\sigma_{iu}t_{u}\left[n\right]+\sum_{\begin{array}{l} v=1 \end{array}}^{V_{i}}\varsigma_{iv}l_{v}\left[n\right]-\sum_{w=1}^{W_{i}}\tau_{iw}m_{w}\left[n\right]l_{i}\left[n\right]+\lambda_{i,LRM}+\mu_{i,LRM}\left[n\right]\\ ,\;\mathrm{for}\;i=1,\ldots ,I,n=1,\ldots ,N \end{array}$$

where $l_{i}\left[n\right]$ means the expression level of the *i*-th lncRNA; $t_{u}\left[n\right]$, $l_{v}\left[n\right]$ and $m_{w}\left[n\right]$ indicate the expression level of the *u*-th TF, the *v*-th lncRNA and the the *w*-th miRNA of the *n*-th sample, respectively; $U_{i}$, $V_{i}$ and $W_{i}$ individually represent the total binding number of TFs, lncRNAs and miRNAs on the *i*-th lncRNAs, respectively; $\sigma_{iu}$ is the transcriptional regulatory ability from the *u*-th TF on the *i*-th lncRNA; $\varsigma_{iv}$ expresses the transcriptional regulatory ability from the *v*-th lncRNA on the *i*-th lncRNA; $\tau_{iw}\geq 0$ denotes the post-transcriptional regulatory ability from the *w*-th miRNA on the *i*-th lncRNA; I denotes the total number of lncRNAs and *N* means the total number of samples; $\lambda_{i,LRM}$ indicates the basal level of the *i*-th lncRNA; $\mu_{i,LRM}\left[n\right]$ is the data noise.

Fourth, the expression of the *j*-th miRNA is also affected by the TFs, lncRNAs and miRNAs. Furthermore, we can illustrate the regulation of miRNA in the miRNA model (MRM) of the candidate GWGEN through the following equation:

$$\begin{array}{l} m_{j}\left[n\right]=\sum_{u=1}^{U_{j}}\omega_{ju}t_{u}\left[n\right]+\sum_{\begin{array}{l} v=1 \end{array}}^{V_{j}}\xi_{jv}l_{v}\left[n\right]-\sum_{w=1}^{W_{j}}\psi_{jw}m_{w}\left[n\right]m_{j}\left[n\right]+\lambda_{j,MRM}+\mu_{j,MRM}\left[n\right]\\ ,\;\mathrm{for}\;j=1,\ldots ,J,n=1,\ldots ,N \end{array}$$

where $m_{j}\left[n\right]$ means the expression level of the *j*-th miRNA; $t_{u}\left[n\right]$, $l_{v}\left[n\right]$ and $m_{w}\left[n\right]$ separately represent the expression level of the *u*-th TF, the *v*-th lncRNA and the *w*-th miRNA, respectively; $U_{j}$, $V_{j}$ and $W_{j}$ are the binding total numbers of TFs, lncRNAs and miRNAs, respectively; $\omega_{ju}$ expresses the transcriptional regulatory ability from the *u*-th TF on the *j*-th miRNA; $\xi_{jv}$ denotes the transcriptional regulatory ability from the *v*-th lncRNA on the *j*-th miRNA; $\psi_{jw}$ denotes the post-transcriptional regulatory ability from the *w*-th miRNA on the *j*-th miRNA; *J* indicates the total number of miRNAs and *N* is the total number of samples; $\lambda_{j.MRM}$ represents the basal level of the *j*-th miRNA; $\mu_{j,MRM}\left[n\right]$ means the data noise.

### 4.4. The System Identification and System Order Detection Methods for Real GWGENs of OSCC and Non-OSCC from the Candidate GWGEN

According to the interaction and regulation models we described above, the candidate PPIN is generated from PPI models in (1); the candidate GRN is described and combined by the corresponding regulation models in (2)–(4). We obtain the real GWGENs of OSCC and non-OSCC through pruning the false positive interactions and regulations by system identification and system order detection methods based on the microarray data of OSCC and non-OSCC, respectively. To identify the parameters of the above interactive and regulatory models, Equations (1)–(4) could be respectively expressed by the following linear regression forms.

(1) $$p_{q}[n]=\left[\begin{array}{ccccc} p_{q}[n]p_{1}[n] & p_{q}[n]p_{2}[n] & \cdots & p_{q}[n]p_{G_{q}}[n] & 1 \end{array}\right]\times \left[\begin{array}{c} \kappa_{q1}\\ \kappa_{q2}\\ \vdots \\ \kappa_{qG_{q}}\\ \lambda_{q,PPIM} \end{array}\right]+\mu_{q,PPIM}[n]$$

(2) $$g_{x}[n]=\left[\begin{array}{cccccccccc} t_{1}[n] & \cdots & t_{U_{x}} & l_{1}[n] & \cdots & l_{V_{x}} & m_{1}[n]g_{x}\left[n\right] & \cdots & m_{W_{x}}[n]g_{x}\left[n\right] & 1 \end{array}\right]\times \left[\begin{array}{c} \alpha_{x1}\\ \vdots \\ \alpha_{xU_{x}}\\ \beta_{1}\\ \vdots \\ \beta_{xV_{x}}\\ -\gamma_{1}\\ \vdots \\ -\gamma_{xWx}\\ \lambda_{x,GRM} \end{array}\right]+\mu_{x,GRM}[n]$$

(3) $$l_{i}[n]=\left[\begin{array}{cccccccccc} t_{1}[n] & \cdots & t_{U_{i}} & l_{1}[n] & \cdots & l_{V_{i}} & m_{1}[n]l_{i}\left[n\right] & \cdots & m_{W_{i}}[n]l_{i}\left[n\right] & 1 \end{array}\right]\times \left[\begin{array}{c} \alpha_{i1}\\ \vdots \\ \alpha_{iU_{i}}\\ \beta_{1}\\ \vdots \\ \beta_{iV_{i}}\\ -\gamma_{1}\\ \vdots \\ -\gamma_{iW_{i}}\\ \lambda_{i,LRM} \end{array}\right]+\mu_{i,LRM}[n]$$

(4) $$m_{j}[n]=\left[\begin{array}{cccccccccc} t_{1}[n] & \cdots & t_{U_{j}} & l_{1}[n] & \cdots & l_{V_{j}} & m_{1}[n]m_{j}\left[n\right] & \cdots & m_{W_{j}}[n]m_{j}\left[n\right] & 1 \end{array}\right]\times \left[\begin{array}{c} \alpha_{j1}\\ \vdots \\ \alpha_{jU_{j}}\\ \beta_{1}\\ \vdots \\ \beta_{jV_{j}}\\ -\gamma_{1}\\ \vdots \\ -\gamma_{jW_{j}}\\ \lambda_{j,MRM} \end{array}\right]+\mu_{j,MRM}[n]$$

for q=1,…,Q, x=1,…,X, i=1,…,I, j=1,…,J, n=1,…,N, where (5)–(8) are individually the regression forms for the protein interactions in PPIN and the regulations in GRN in the candidate GWGEN. Q, X, I and J are, respectively, the total number of proteins, genes, lncRNAs and miRNAs in the candidate GWGWN, and N means the total number of samples.

The linear regression equations in (5)–(8) could be simply expressed as the following equations:

(5) $$p_{q}\left[n\right]=\phi_{q,PPIM}\left[n\right]\cdot \theta_{q,PPIM}+\varepsilon_{q,PPIM},\;\mathrm{for}\;q=1,\ldots ,Q$$

(6) $$g_{x}\left[n\right]=\phi_{x,GRM}\left[n\right]\cdot \theta_{x,GRM}+\varepsilon_{x,GRM},\;\mathrm{for}\;x=1,\ldots ,X$$

(7) $$l_{i}\left[n\right]=\phi_{i,LRM}\left[n\right]\cdot \theta_{i,LRM}+\varepsilon_{i,LRM},\;\mathrm{for}\;i=1,\ldots ,I$$

(8) $$m_{j}\left[n\right]=\phi_{j,MRM}\left[n\right]\cdot \theta_{j,MRM}+\varepsilon_{j,MRM},\;\mathrm{for}\;j=1,\ldots ,J$$

where $\phi_{q,PPIM}\left[n\right]$, $\phi_{x,GRM}\left[n\right]$, $\phi_{i,LRM}\left[n\right]$ and $\phi_{j,MRM}\left[n\right]$ individually mean the regression vectors of proteins, genes, lncRNAs and miRNAs in the candidate GWGEN in the *n*-th sample; $\theta_{q,PPIM}$ is the parameter vector of the protein–protein interaction abilities and the protein basal levels; $\theta_{x,GRM}$, $\theta_{i,LRM}$ and $\theta_{j,MRM}$ respectively express the parameter vectors of the transcriptional regulatory abilities and basal levels of the genes, lncRNAs and miRNAs; $\varepsilon_{q,PPIM}$, $\varepsilon_{x,GRM}$, $\varepsilon_{i,LRM}$ and $\varepsilon_{j,MRM}$ are separately the noises for protein interactions and regulations in the candidate GWGEN.

The above linear regression forms for *N* samples are denoted, respectively, as the following:

(9) $$\left[\begin{array}{c} p_{q}\left[1\right]\\ p_{q}\left[2\right]\\ \vdots \\ p_{q}\left[N\right] \end{array}\right]=\left[\begin{array}{c} \phi_{q,PPIM}\left[1\right]\\ \phi_{q,PPIM}\left[2\right]\\ \vdots \\ \phi_{q,PPIM}\left[N\right] \end{array}\right]\cdot \theta_{q,PPIM}+\left[\begin{array}{c} \varepsilon_{q,PPIM}\left[1\right]\\ \varepsilon_{q,PPIM}\left[2\right]\\ \vdots \\ \varepsilon_{q,PPIM}\left[N\right] \end{array}\right],\;\mathrm{for}\;q=1,\ldots ,Q$$

(10) $$\left[\begin{array}{c} g_{x}\left[1\right]\\ g_{x}\left[2\right]\\ \vdots \\ g_{x}\left[N\right] \end{array}\right]=\left[\begin{array}{c} \phi_{x,GRM}\left[1\right]\\ \phi_{x,GRM}\left[2\right]\\ \vdots \\ \phi_{x,GRM}\left[N\right] \end{array}\right]\cdot \theta_{x,GRM}+\left[\begin{array}{c} \varepsilon_{x,GRM}\left[1\right]\\ \varepsilon_{x,GRM}\left[2\right]\\ \vdots \\ \varepsilon_{x,GRM}\left[N\right] \end{array}\right],\;\mathrm{for}\;x=1,\ldots ,X$$

(11) $$\left[\begin{array}{c} l_{i}\left[1\right]\\ l_{i}\left[2\right]\\ \vdots \\ l_{i}\left[N\right] \end{array}\right]=\left[\begin{array}{c} \phi_{i,LRM}\left[1\right]\\ \phi_{i,LRM}\left[2\right]\\ \vdots \\ \phi_{i,LRM}\left[N\right] \end{array}\right]\cdot \theta_{i,LRM}+\left[\begin{array}{c} \varepsilon_{i,LRM}\left[1\right]\\ \varepsilon_{i,LRM}\left[2\right]\\ \vdots \\ \varepsilon_{i,LRM}\left[N\right] \end{array}\right],\;\mathrm{for}\;i=1,\ldots ,I$$

(12) $$\left[\begin{array}{c} m_{j}\left[1\right]\\ m_{j}\left[2\right]\\ \vdots \\ m_{j}\left[N\right] \end{array}\right]=\left[\begin{array}{c} \phi_{j,MRM}\left[1\right]\\ \phi_{j,MRM}\left[2\right]\\ \vdots \\ \phi_{j,MRM}\left[N\right] \end{array}\right]\cdot \theta_{j,MRM}+\left[\begin{array}{c} \varepsilon_{j,MRM}\left[1\right]\\ \varepsilon_{j,MRM}\left[2\right]\\ \vdots \\ \varepsilon_{j,MRM}\left[N\right] \end{array}\right],\;\mathrm{for}\;j=1,\ldots ,J$$

The above equations could be individually expressed as the following algebraic equations:

(13) $$P_{q}=\Phi_{q,PPIM}\cdot \Theta_{q,PPIM}+E_{q,PPIM},\;\mathrm{for}\;q=1,\ldots ,Q$$

(14) $$G_{x}=\Phi_{x,GRM}\cdot \Theta_{x,GRM}+E_{x,GRM},\;\mathrm{for}\;x=1,\ldots ,X$$

(15) $$L_{i}=\Phi_{i,LRM}\cdot \Theta_{i,LRM}+E_{i,LRM},\;\mathrm{for}\;i=1,\ldots ,I$$

(16) $$M_{j}=\Phi_{j,MRM}\cdot \Theta_{j,MRM}+E_{j,MRM},\;\mathrm{for}\;j=1,\ldots ,J$$

where $\Phi_{q,PPIM}$, $\Phi_{x,GRM}$, $\Phi_{i,LRM}$ and $\Phi_{j,MRM}$ separately express the regression matrix of proteins, genes, lncRNAs and miRNAs of N samples. $\Theta_{q,PPIM}$, $\Theta_{x,GRM}$, $\Theta_{i,LRM}$ and $\Theta_{j,MRM}$ individually mean the corresponding interactive and regulatory parameter vectors. ${Ε}_{q,PPIM}$, ${Ε}_{x,GRM}$, ${Ε}_{i,LRM}$ and ${Ε}_{j,MRM}$ respectively denote the corresponding data noise vectors.

To identify the interactive and regulatory parameter of the candidate GWGEN, we estimate the parameter vectors $\theta_{q,PPIM}$, $\theta_{q,GRM}$, $\theta_{q,LRM}$ and $\theta_{q,MRM}$ by the least square method with the negative regulation constraint on miRNA, as follows:

(17) $${\hat{\Theta }}_{q,PPIM}=\underset{\Theta_{q,PPIM}}{\mathrm{argmin}}\frac{1}{2}{\left\|\Phi_{q,PPIM}\cdot \Theta_{q,PPIM}-P_{q}\right\|}_{2}^{2}$$

(18) $$\begin{array}{l} {\hat{\Theta }}_{x,GRM}=\underset{\Theta_{x,GRM}}{\mathrm{argmin}}\frac{1}{2}{\left\|\Phi_{x,GRM}\cdot \Theta_{x,GRM}-G_{x}\right\|}_{2}^{2}\\ \mathrm{subject}\;\mathrm{to}\;\left[\begin{array}{cccc} \underset{U_{i}}{\underset{︸}{\begin{array}{ccccc} 0 & 0 & \cdots & \cdots & 0\\ 0 & 0 & \cdots & \cdots & 0\\ \vdots & \vdots & \ddots & \ddots & \vdots \\ \vdots & \vdots & \ddots & \ddots & \vdots \\ 0 & 0 & \cdots & \cdots & 0 \end{array}}}| & \underset{V_{i}}{\underset{︸}{\begin{array}{ccccc} 0 & 0 & \cdots & \cdots & 0\\ 0 & 0 & \cdots & \cdots & 0\\ \vdots & \vdots & \ddots & \ddots & \vdots \\ \vdots & \vdots & \ddots & \ddots & \vdots \\ 0 & 0 & \cdots & \cdots & 0 \end{array}}}| & \underset{W_{i}}{\underset{︸}{\begin{array}{ccccc} 1 & 0 & \cdots & \cdots & 0\\ 0 & 1 & \cdots & \cdots & 0\\ \vdots & \vdots & \ddots & \ddots & \vdots \\ 0 & 0 & \ddots & 1 & 0\\ 0 & 0 & \cdots & 0 & 1 \end{array}}}| & \begin{array}{c} 0\\ 0\\ \vdots \\ \vdots \\ 0 \end{array} \end{array}\right]\Theta_{i,GRM}\leq \left[\begin{array}{c} 0\\ 0\\ \vdots \\ \vdots \\ 0 \end{array}\right] \end{array}$$

(19) $$\begin{array}{l} {\hat{\Theta }}_{i,LRM}=\underset{\Theta_{i,LRM}}{\mathrm{argmin}}\frac{1}{2}{\left\|\Phi_{i,LRM}\cdot \Theta_{i,LRM}-L_{i}\right\|}_{2}^{2}\\ \mathrm{subject}\;\mathrm{to}\;\left[\begin{array}{cccc} \underset{U_{i}}{\underset{︸}{\begin{array}{ccccc} 0 & 0 & \cdots & \cdots & 0\\ 0 & 0 & \cdots & \cdots & 0\\ \vdots & \vdots & \ddots & \ddots & \vdots \\ \vdots & \vdots & \ddots & \ddots & \vdots \\ 0 & 0 & \cdots & \cdots & 0 \end{array}}}| & \underset{V_{i}}{\underset{︸}{\begin{array}{ccccc} 0 & 0 & \cdots & \cdots & 0\\ 0 & 0 & \cdots & \cdots & 0\\ \vdots & \vdots & \ddots & \ddots & \vdots \\ \vdots & \vdots & \ddots & \ddots & \vdots \\ 0 & 0 & \cdots & \cdots & 0 \end{array}}}| & \underset{W_{i}}{\underset{︸}{\begin{array}{ccccc} 1 & 0 & \cdots & \cdots & 0\\ 0 & 1 & \cdots & \cdots & 0\\ \vdots & \vdots & \ddots & \ddots & \vdots \\ 0 & 0 & \ddots & 1 & 0\\ 0 & 0 & \cdots & 0 & 1 \end{array}}}| & \begin{array}{c} 0\\ 0\\ \vdots \\ \vdots \\ 0 \end{array} \end{array}\right]\Theta_{i,LRM}\leq \left[\begin{array}{c} 0\\ 0\\ \vdots \\ \vdots \\ 0 \end{array}\right] \end{array}$$

(20) $$\begin{array}{l} {\hat{\Theta }}_{j,MRM}=\underset{\Theta_{j,MRM}}{\mathrm{argmin}}\frac{1}{2}{\left\|\Phi_{j,MRM}\cdot \Theta_{j,MRM}-M_{j}\right\|}_{2}^{2}\\ \mathrm{subject}\;\mathrm{to}\;\left[\begin{array}{cccc} \underset{U_{j}}{\underset{︸}{\begin{array}{ccccc} 0 & 0 & \cdots & \cdots & 0\\ 0 & 0 & \cdots & \cdots & 0\\ \vdots & \vdots & \ddots & \ddots & \vdots \\ \vdots & \vdots & \ddots & \ddots & \vdots \\ 0 & 0 & \cdots & \cdots & 0 \end{array}}}| & \underset{V_{j}}{\underset{︸}{\begin{array}{ccccc} 0 & 0 & \cdots & \cdots & 0\\ 0 & 0 & \cdots & \cdots & 0\\ \vdots & \vdots & \ddots & \ddots & \vdots \\ \vdots & \vdots & \ddots & \ddots & \vdots \\ 0 & 0 & \cdots & \cdots & 0 \end{array}}}| & \underset{W_{j}}{\underset{︸}{\begin{array}{ccccc} 1 & 0 & \cdots & \cdots & 0\\ 0 & 1 & \cdots & \cdots & 0\\ \vdots & \vdots & \ddots & \ddots & \vdots \\ 0 & 0 & \ddots & 1 & 0\\ 0 & 0 & \cdots & 0 & 1 \end{array}}}| & \begin{array}{c} 0\\ 0\\ \vdots \\ \vdots \\ 0 \end{array} \end{array}\right]\Theta_{j,MRM}\leq \left[\begin{array}{c} 0\\ 0\\ \vdots \\ \vdots \\ 0 \end{array}\right] \end{array}$$

For the constrained optimization problems for the parameter estimation problems (21)–(24), we look for the optimal parameter estimates of the candidate GWGEN through the microarray data of OSCC and non-OSCC as follows: interactive parameters between proteins ${\hat{\Theta }}_{q,PPIM}$, the regulatory parameters of genes ${\hat{\Theta }}_{x,GRM}$, lncRNAs ${\hat{\Theta }}_{i,LRM}$ and miRNAs ${\hat{\Theta }}_{j,MRM}$. These constrained optimization problems for the parameter estimation of the candidate GWGEN were solved by the MATLAB Optimization Toolbox. It is worth noting that the negative inequality constraints in (22)–(24) represent that the regulatory parameters of miRNAs should be less than or equal to zero to ensure the negative regulation of miRNAs on genes, lncRNAs and miRNAs.

After the parameter estimation of the candidate GWGEN of non-OSCC and OSCC by the respective microarray data, we used the system order detection method, AIC, to detect the system order (the number of interactions of each protein or the number of regulations of each gene, lncRNA and miRNA). The equations of AIC for each protein, gene, lncRNA and miRNA are given as follows.

(21) $$\begin{array}{l} AIC(Q_{q})=\log(\Omega_{q,PPIM})+\frac{2(G_{q}+1)}{N},\;\mathrm{for}\;q=1,\ldots ,Q\\ \mathrm{where}\Omega_{q,PPIM}=\frac{{\left(P_{q}-\Phi_{q,PPIM}\cdot {\hat{\Theta }}_{q,PPIM}\right)}^{T}(P_{q}-\Phi_{q,PPIM}\cdot {\hat{\Theta }}_{q,PPIM})}{N} \end{array}$$

where $\Omega_{q,PPIM}$ is the estimated residual error of the *q*-th protein from the least square parameter estimation ${\hat{\Theta }}_{q,PPIM}$ in (21), and $Q_{q}$ means the number of protein interactions with the *q*-th protein.

(22) $$\begin{array}{l} AIC(U_{x},V_{x},W_{x})=\log(\Omega_{x,GRM})+\frac{2(O_{x,GRM}+1)}{N},\;\mathrm{for}\;x=1,\ldots ,X\\ \mathrm{where}\;\Omega_{x,GRM}=\frac{{\left(G_{x}-\Phi_{x,GRM}\cdot {\hat{\Theta }}_{x,GRM}\right)}^{T}(G_{x}-\Phi_{x,GRM}\cdot {\hat{\Theta }}_{x,GRM})}{N},O_{x,GRM}=U_{x}+V_{x}+W_{x} \end{array}$$

where $\Omega_{x,GRM}$ means the estimated residual error of the *x*-th gene in (22), and $O_{x,GRM}$ is the number of regulations of the genes, lncRNAs and miRNAs on the *x*-th gene; ${\hat{\Theta }}_{x,GRM}$ denotes the estimated parameters in (22).

(23) $$\begin{array}{l} AIC(U_{i},V_{i},W_{i})=\log(\Omega_{i,LRM})+\frac{2(O_{i,LRM}+1)}{N},\;\mathrm{for}\;i=1,\ldots ,I\\ \mathrm{where}\;\Omega_{i,LRM}=\frac{{\left(L_{i}-\Phi_{i,LRM}\cdot {\hat{\Theta }}_{i,LRM}\right)}^{T}(L_{i}-\Phi_{i,LRM}\cdot {\hat{\Theta }}_{i,LRM})}{N},O_{i,LRM}=U_{i}+V_{i}+W_{i} \end{array}$$

where $\Omega_{i,LRM}$ denotes the estimated residual error of the *i*-th lncRNA in (23), and $O_{i,LRM}$ is the number of regulations of the genes, lncRNAs and miRNAs on the *i*-th lncRNA; ${\hat{\Theta }}_{i,LRM}$ indicates the estimated parameters in (23).

(24) $$\begin{array}{l} AIC(U_{j},V_{j},W_{j})=\log(\Omega_{j,MRM})+\frac{2(O_{j,MRM}+1)}{N},\;\mathrm{for}\;j=1,\ldots ,J\\ \mathrm{where}\;\Omega_{j,MRM}=\frac{{\left(M_{j}-\Phi_{j,MRM}\cdot {\hat{\Theta }}_{j,MRM}\right)}^{T}(M_{j}-\Phi_{j,MRM}\cdot {\hat{\Theta }}_{j,MRM})}{N},O_{j,MRM}=U_{j}+V_{j}+W_{j} \end{array}$$

where $\Omega_{j.MRM}$ denotes the estimated residual error of the *j*-th miRNA in (24), and $O_{j.MRM}$ indicates the number of regulations of the genes, lncRNAs and miRNAs on the *j*-th miRNA; ${\hat{\Theta }}_{j,MRM}$ denotes the estimated parameters in (24).

The AIC system order detection method in (25) means that if the system order (number interactions) $Q_{q}$ increases, the second term of AIC in (25) will increase and the first term of AIC will decrease, and vice versa. The real number of interactions (system order) $Q_{q}$* will balance two terms and achieve the minimum AIC ($Q_{q}$). Therefore, employ the AIC technique to determine the real number of interactions or regulations for each protein, gene, miRNA and lncRNA by their AICs in (25)–(28) in the candidate GWGEN to prune the false positive interactions and regulations and obtain the real GWGENs of OSCC and non-OSCC in the following.

Considering the order detection method of AIC in system identification, the real order of the system (i.e., the total number of interactions of the *q*-th protein in (1) or the number of regulations on the *x*-th in (2)) is to minimize the AIC problems of system identification in (21)–(28). Therefore, the real number of interactions or regulations for every protein, gene, lncRNA and miRNA in the candidate GWGEN can be obtained by solving the AIC minimization problems below:

(25) $$Q_{q}^{*}=\underset{Q_{q}}{\mathrm{argmin}}AIC(G_{q}),\;\mathrm{for}\;q=1,\ldots ,Q$$

(26) $$(U_{x}^{*},V_{x}^{*},W_{x}^{*})=\underset{U_{x},V_{x},W_{x}}{\mathrm{argmin}}AIC(U_{x},V_{x},W_{x}),\;\mathrm{for}\;x=1,\ldots ,X$$

(27) $$(U_{i}^{*},V_{i}^{*},W_{i}^{*})=\underset{U_{i},V_{i},W_{i}}{\mathrm{argmin}}AIC(U_{i},V_{i},W_{i}),\;\mathrm{for}\;i=1,\ldots ,I$$

(28) $$(U_{j}^{*},V_{j}^{*},W_{j}^{*})=\underset{U_{j},V_{j},W_{j}}{\mathrm{argmin}}AIC(U_{j},V_{j},W_{j}),\;\mathrm{for}\;j=1,\ldots ,J$$

where $Q_{q}^{*}$ means the real number of protein interactions for the *q*-th protein; $U_{x}^{*},V_{x}^{*}\;\mathrm{and}\;W_{x}^{*}$ respectively express the real number of regulations of genes, lncRNAs and miRNAs on the *x*-th gene; $U_{i}^{*},V_{i}^{*}\;\mathrm{and}\;W_{i}^{*}$ represent the real number of regulations of genes, lncRNAs and miRNAs on the *i*-th lncRNA, respectively; $U_{j}^{*},V_{j}^{*}\;\mathrm{and}\;W_{j}^{*}$ are individually the real number of regulations of genes, lncRNAs and miRNAs on the *j*-th miRNA. Therefore, the interactions of proteins and the regulations of the gene, miRNA and lncRNA, which are out of real order by solving the AIC minimization problems in (29)–(32), are thought of as false positives in the candidate GWGEN of non-OSCC and OSCC and should be pruned out to obtain the real GWGENs of non-OSCC and OSCC in Figure 2, respectively.

### 4.5. The Principal Network Projection (PNP) Method for the Core GWGENs by Extracting from Real GWGENs

We want to compare the signaling pathways of non-OSCC and OSCC to investigate their genetic and epigenetic carcinogenic molecular mechanism. Therefore, we need to transform real GWGENs of non-OSCC and OSCC to signaling pathways of non-OSCC and OSCC, respectively, by the annotation of KEGG pathways. However, at present, only a GWGEN with 6000 nodes at most can be annotated by KEGG signal pathways. Therefore, the principal network projection (PNP) method on the basis of the singular value decomposition (SVD) is employed to extract the core GWGENs with 6000 nodes from the real GWGENs for the annotation of core signaling pathways by KEGG pathways. Before we extract the core GWGENs, a network matrix H of real GWGENs should be introduced. The network matrix H consists of interactions among proteins and regulations of the TF-gene, TF-lncRNA, TF-miRNA, lncRNA-gene, lncRNA-lncRNA, lncRNA-miRNA, miRNA-gene, miRNA-lncRNA and miRNA-miRNA in real GWGENs, as follows:

(29) $$H=\left[\begin{array}{rrr} h_{protein\Leftrightarrow protein} & 0 & 0\\ h_{TF\Rightarrow \mathrm{gene}} & h_{\ln cRNA\Rightarrow gene} & h_{miRNA\Rightarrow gene}\\ h_{TF\Rightarrow \ln cRNA} & h_{\ln cRNA\Rightarrow \ln cRNA} & h_{miRNA\Rightarrow \ln cRNA}\\ h_{TF\Rightarrow \mathrm{mi}RNA} & h_{\ln cRNA\Rightarrow miRNA} & h_{miRNA\Rightarrow miRNA} \end{array}\right]$$

where $h_{protein\Leftrightarrow protein}$ is the sub-matrix of PPIs, of which the bidirectional arrow at the subscript of the sub-matrix means that the protein interaction is bidirectional; $h_{TF\Rightarrow \mathrm{gene}}$, $h_{TF\Rightarrow \ln cRNA}$, $h_{TF\Rightarrow \mathrm{mi}RNA}$, $h_{\ln cRNA\Rightarrow gene}$, $h_{\ln cRNA\Rightarrow \ln cRNA}$, $h_{\ln cRNA\Rightarrow miRNA}$, $h_{miRNA\Rightarrow gene}$, $h_{miRNA\Rightarrow \ln cRNA}$ and $h_{miRNA\Rightarrow miRNA}$ represent the transcriptional regulatory sub-matrixes of TFs on genes, lncRNAs and miRNAs; lncRNAs on genes, lncRNAs and miRNAs; and miRNAs on genes, lncRNAs and miRNAs, respectively. The network matrix H of real GWGENs is given in detail, as follows:

(30) $$H=\left[\begin{array}{rrrrrrrrrrrrrrrrrr} {\hat{\kappa }}_{11} & {\hat{\kappa }}_{12} & \cdots & {\hat{\kappa }}_{1r} & \cdots & {\hat{\kappa }}_{1Q_{q}} & 0 & 0 & \cdots & 0 & \cdots & 0 & 0 & 0 & \cdots & 0 & \cdots & 0\\ {\hat{\kappa }}_{21} & {\hat{\kappa }}_{22} & \cdots & {\hat{\kappa }}_{2r} & \cdots & {\hat{\kappa }}_{2Q_{q}} & 0 & 0 & \cdots & 0 & \cdots & 0 & 0 & 0 & \cdots & 0 & \cdots & 0\\ \vdots & \vdots & \ddots & \vdots & \ddots & \vdots & \vdots & \vdots & \ddots & \vdots & \ddots & \vdots & \vdots & \vdots & \ddots & \vdots & \ddots & \vdots \\ {\hat{\kappa }}_{q1} & {\hat{\kappa }}_{q2} & \cdots & {\hat{\kappa }}_{qr} & \cdots & {\hat{\kappa }}_{qQ_{q}} & 0 & 0 & \cdots & 0 & \cdots & 0 & 0 & 0 & \cdots & 0 & \cdots & 0\\ \vdots & \vdots & \ddots & \vdots & \ddots & \vdots & \vdots & \vdots & \ddots & \vdots & \ddots & \vdots & \vdots & \vdots & \ddots & \vdots & \ddots & \vdots \\ {\hat{\kappa }}_{Q1} & {\hat{\kappa }}_{Q1} & \cdots & {\hat{\kappa }}_{Q1} & \cdots & {\hat{\kappa }}_{QQ_{q}} & 0 & 0 & \cdots & 0 & \cdots & 0 & 0 & 0 & \cdots & 0 & \cdots & 0\\ {\hat{\alpha }}_{11} & {\hat{\alpha }}_{12} & \cdots & {\hat{\alpha }}_{1u} & \cdots & {\hat{\alpha }}_{1U_{x}} & {\hat{\beta }}_{11} & {\hat{\beta }}_{12} & \cdots & {\hat{\beta }}_{1v} & \cdots & {\hat{\beta }}_{1V_{x}} & {\hat{\gamma }}_{11} & {\hat{\gamma }}_{12} & \cdots & {\hat{\gamma }}_{1w} & \cdots & {\hat{\gamma }}_{1W_{x}}\\ {\hat{\alpha }}_{21} & {\hat{\alpha }}_{22} & \cdots & {\hat{\alpha }}_{2u} & \cdots & {\hat{\alpha }}_{2U_{x}} & {\hat{\beta }}_{21} & {\hat{\beta }}_{22} & \cdots & {\hat{\beta }}_{2v} & \cdots & {\hat{\beta }}_{2V_{x}} & {\hat{\omega }}_{21} & {\hat{\omega }}_{22} & \cdots & {\hat{\gamma }}_{2w} & \cdots & {\hat{\gamma }}_{2W_{x}}\\ \vdots & \vdots & \ddots & \vdots & \ddots & \vdots & \vdots & \vdots & \ddots & \vdots & \ddots & \vdots & \vdots & \vdots & \ddots & \vdots & \ddots & \vdots \\ {\hat{\alpha }}_{x1} & {\hat{\alpha }}_{x2} & \cdots & {\hat{\alpha }}_{xu} & \cdots & {\hat{\alpha }}_{xU_{x}} & {\hat{\beta }}_{x1} & {\hat{\beta }}_{x2} & \cdots & {\hat{\beta }}_{xv} & \cdots & {\hat{\beta }}_{xV_{x}} & {\hat{\gamma }}_{x1} & {\hat{\gamma }}_{x2} & \cdots & {\hat{\gamma }}_{xw} & \cdots & {\hat{\gamma }}_{xW_{x}}\\ \vdots & \vdots & \ddots & \vdots & \ddots & \vdots & \vdots & \vdots & \ddots & \vdots & \ddots & \vdots & \vdots & \vdots & \ddots & \vdots & \ddots & \vdots \\ {\hat{\alpha }}_{X1} & {\hat{\alpha }}_{X2} & \cdots & {\hat{\alpha }}_{Xu} & \cdots & {\hat{\alpha }}_{XU_{x}} & {\hat{\beta }}_{X1} & {\hat{\beta }}_{X2} & \cdots & {\hat{\beta }}_{Xv} & \cdots & {\hat{\beta }}_{XV_{x}} & {\hat{\gamma }}_{X1} & {\hat{\gamma }}_{X2} & \cdots & {\hat{\gamma }}_{Xw} & \cdots & {\hat{\gamma }}_{XW_{x}}\\ {\hat{\sigma }}_{11} & {\hat{\sigma }}_{12} & \cdots & {\hat{\sigma }}_{1u} & \cdots & {\hat{\sigma }}_{1U_{i}} & {\hat{\varsigma }}_{11} & {\hat{\varsigma }}_{12} & \cdots & {\hat{\varsigma }}_{1v} & \cdots & {\hat{\varsigma }}_{1V_{i}} & {\hat{\tau }}_{11} & {\hat{\tau }}_{12} & \cdots & {\hat{\tau }}_{1w} & \cdots & {\hat{\tau }}_{1W_{i}}\\ {\hat{\sigma }}_{21} & {\hat{\sigma }}_{22} & \cdots & {\hat{\sigma }}_{2u} & \cdots & {\hat{\sigma }}_{2U_{i}} & {\hat{\varsigma }}_{21} & {\hat{\varsigma }}_{22} & \cdots & {\hat{\varsigma }}_{2v} & \cdots & {\hat{\varsigma }}_{2V_{i}} & {\hat{\tau }}_{21} & {\hat{\tau }}_{22} & \cdots & {\hat{\tau }}_{2w} & \cdots & {\hat{\tau }}_{2W_{i}}\\ \vdots & \vdots & \ddots & \vdots & \ddots & \vdots & \vdots & \vdots & \ddots & \vdots & \ddots & \vdots & \vdots & \vdots & \ddots & \vdots & \ddots & \vdots \\ {\hat{\sigma }}_{i1} & {\hat{\sigma }}_{i2} & \cdots & {\hat{\sigma }}_{iu} & \cdots & {\hat{\sigma }}_{iU_{i}} & {\hat{\varsigma }}_{i1} & {\hat{\varsigma }}_{i2} & \cdots & {\hat{\varsigma }}_{iv} & \cdots & {\hat{\varsigma }}_{iV_{i}} & {\hat{\tau }}_{i1} & {\hat{\tau }}_{i2} & \cdots & {\hat{\tau }}_{iw} & \cdots & {\hat{\tau }}_{iW_{i}}\\ \vdots & \vdots & \ddots & \vdots & \ddots & \vdots & \vdots & \vdots & \ddots & \vdots & \ddots & \vdots & \vdots & \vdots & \ddots & \vdots & \ddots & \vdots \\ {\hat{\sigma }}_{I1} & {\hat{\sigma }}_{I2} & \cdots & {\hat{\sigma }}_{Iu} & \cdots & {\hat{\sigma }}_{IU_{i}} & {\hat{\varsigma }}_{I1} & {\hat{\varsigma }}_{I2} & \cdots & {\hat{\varsigma }}_{Iv} & \cdots & {\hat{\varsigma }}_{IV_{i}} & {\hat{\tau }}_{I1} & {\hat{\tau }}_{I2} & \cdots & {\hat{\tau }}_{Iw} & \cdots & {\hat{\tau }}_{IW_{i}}\\ {\hat{\omega }}_{11} & {\hat{\omega }}_{12} & \cdots & {\hat{\omega }}_{1u} & \cdots & {\hat{\omega }}_{1U_{j}} & {\hat{\xi }}_{11} & {\hat{\xi }}_{12} & \cdots & {\hat{\xi }}_{1v} & \cdots & {\hat{\xi }}_{1U_{j}} & {\hat{\psi }}_{11} & {\hat{\psi }}_{12} & \cdots & {\hat{\psi }}_{1w} & \cdots & {\hat{\psi }}_{1W_{j}}\\ {\hat{\omega }}_{21} & {\hat{\omega }}_{22} & \cdots & {\hat{\omega }}_{2u} & \cdots & {\hat{\omega }}_{2U_{j}} & {\hat{\xi }}_{21} & {\hat{\xi }}_{22} & \cdots & {\hat{\xi }}_{2v} & \cdots & {\hat{\xi }}_{2U_{j}} & {\hat{\psi }}_{21} & {\hat{\psi }}_{22} & \cdots & {\hat{\psi }}_{2w} & \cdots & {\hat{\psi }}_{2W_{j}}\\ \vdots & \vdots & \ddots & \vdots & \ddots & \vdots & \vdots & \vdots & \ddots & \vdots & \ddots & \vdots & \vdots & \vdots & \ddots & \vdots & \ddots & \vdots \\ {\hat{\omega }}_{j1} & {\hat{\omega }}_{j2} & \cdots & {\hat{\omega }}_{ju} & \cdots & {\hat{\omega }}_{jU_{j}} & {\hat{\xi }}_{j1} & {\hat{\xi }}_{j2} & \cdots & {\hat{\xi }}_{jv} & \cdots & {\hat{\xi }}_{jU_{j}} & {\hat{\psi }}_{j1} & {\hat{\psi }}_{j2} & \cdots & {\hat{\psi }}_{jw} & \cdots & {\hat{\psi }}_{jW_{j}}\\ \vdots & \vdots & \ddots & \vdots & \ddots & \vdots & \vdots & \vdots & \ddots & \vdots & \ddots & \vdots & \vdots & \vdots & \ddots & \vdots & \ddots & \vdots \\ {\hat{\omega }}_{J1} & {\hat{\omega }}_{J2} & \cdots & {\hat{\omega }}_{Ju} & \cdots & {\hat{\omega }}_{JU_{j}} & {\hat{\xi }}_{J1} & {\hat{\xi }}_{J2} & \cdots & {\hat{\xi }}_{Jv} & \cdots & {\hat{\xi }}_{JU_{j}} & {\hat{\psi }}_{J1} & {\hat{\psi }}_{J2} & \cdots & {\hat{\psi }}_{Jw} & \cdots & {\hat{\psi }}_{JW_{j}} \end{array}\right]\in {\mathbb{R}}^{\left(Q^{*}+X^{*}+I^{*}+J^{*})\times (U^{*}+V^{*}+W^{*}\right)}$$

where ${\hat{\kappa }}_{qr}$ denotes the interaction ability between the *q*-th protein and the *r*-th protein; ${\hat{\alpha }}_{xu}$, ${\hat{\beta }}_{xv}$ and ${\hat{\gamma }}_{xw}$ are respectively the regulation abilities of the *u*-th TF on the *x*-th gene, the *v*-th lncRNA on the *x*-th gene and the *w*-th miRNA on the *x*-th gene; ${\hat{\sigma }}_{iu}$, ${\hat{\varsigma }}_{iv}$ and ${\hat{\tau }}_{iw}$ individually express the regulation abilities of the *u*-th TF on the *i*-th lncRNA, the *v*-th lncRNA on the *i*-th lncRNA and the *w*-th miRNA on the *i*-th lncRNA; ${\hat{\omega }}_{ju}$, ${\hat{\xi }}_{jv}$ and ${\hat{\psi }}_{jw}$ respectively mean the regulation abilities of the *u*-th TF on the *j*-th miRNA, the *v*-th lncRNA on the *j*-th miRNA and the *w*-th miRNA on the *w*-th miRNA. Moreover, if there is neither interaction nor regulation between the source and the target, some zeros should be padded in the network matrix in (34). Then, the core GWGENs were obtained by using PNP on the network matrix H with a significant energy threshold of 85%. First, the network matrix H is decomposed by singular value decomposition (SVD), as follows [11,83]:

(31) $$H=SVD^{T}$$

where $S\in {\mathbb{R}}^{\left(Q^{*}+X^{*}+I^{*}+J^{*}\right)\times \left(Q^{*}+X^{*}+I^{*}+J^{*}\right)}$ and $D^{T}\in {\mathbb{R}}^{\left(U^{*}+V^{*}+W^{*}\right)\times \left(U^{*}+V^{*}+W^{*}\right)}$ are unitary singular matrices; $V=diag\left(v_{1},\cdots ,v_{i},\cdots v_{U^{*}+V^{*}+W^{*}}\right)\in {\mathbb{R}}^{\left(Q^{*}+X^{*}+I^{*}+J^{*}\right)\times \left(U^{*}+V^{*}+W^{*}\right)}$ means the diagonal matrix of which the components at the diagonal are the singular values of H and are arranged in descending order, i.e., $v_{1}\geq v_{2}\geq \cdots \geq v_{i}\geq \cdots \geq v_{U^{*}+V^{*}+W^{*}}\geq 0$.

(32) $$V=\left[\begin{array}{cccccc} v_{1} & 0 & \cdots & 0 & \cdots & 0\\ 0 & v_{2} & \cdots & 0 & \cdots & 0\\ \vdots & \vdots & \ddots & \vdots & \ddots & \vdots \\ 0 & 0 & \cdots & v_{i} & \cdots & 0\\ \vdots & \vdots & \ddots & \vdots & \ddots & \vdots \\ 0 & 0 & \cdots & 0 & \cdots & v_{U^{*}+V^{*}+W^{*}}\\ 0 & 0 & \cdots & 0 & \cdots & 0\\ \vdots & \vdots & \ddots & \vdots & \ddots & \vdots \\ 0 & 0 & \cdots & 0 & \cdots & 0 \end{array}\right]$$

Moreover, the normalization of singular values in (36) is defined as follows.

(33) $$E_{i}=\frac{v_{i}^{2}}{\sum_{i=1}^{U^{*}+V^{*}+W^{*}}v_{i}^{2}}\;\mathrm{and}\;\sum_{i=1}^{U^{*}+V^{*}+W^{*}}E_{i}=1$$

(34) $$\sum_{i=1}^{I}E_{i}\geq 0.85$$

From the above equation, we chose the top I significant singular vector structures to indicate the significant energy of real GWGENs, which is equal or more than the threshold of 0.85. Then, we separately projected every node of real GWGENs (i.e., each row of the network matrix H) to the top I significant singular vectors, as follows.

(35) $$Z(a,b)=h_{a,:}\cdot d_{b,:}^{T},\;\mathrm{for}\;a=1,\ldots ,Q^{*}+X^{*}+I^{*}+J^{*},\;b=1,\ldots ,I$$

where Z(a,b) means the projection value of the a-th node on the b-th significant singular vector; *h_a,;_* is the *a*-th row vector of the network matrix H and $d_{:,b}^{T}$ expresses the *b*-th column of $D^{T}$, i.e., the transpose of the *b*-th singular vector. Then, we define the two-norm projection value of each node such as protein, gene, lnRNA and miRNA in real GWGENs on the top I significant singular vectors as follows:

(36) $$S(a)=\sqrt{\sum_{i=1}^{I}Z^{2}(a,b)},\;\mathrm{for}\;a=1,\ldots ,Q^{*}+X^{*}+I^{*}+J^{*}$$

According to the two-norm projection values in (40), the top 6000 significant proteins, genes, miRNAs and lncRNAs with high projection values were chosen to comprise the core GWGENs for OSCC and non-OSCC in Figure 3. Then, the core GWGENs were submitted to the Database for Annotation, Visualization and Integrated Discovery (DAVID) website to conduct the KEGG pathway enrichment analysis in Table 3 and Table 4, and the core signaling pathways of non-OSCC and OSCC in Figure 4 were obtained by exploring the annotation of KEGG pathways. The enrichment analysis was used to check which significant pathways of our results were important to OSCC. Finally, the potential biomarkers were identified in Table 5 for the OSCC carcinogenic mechanism investigated by comparing the core signaling pathways and their downstream abnormal cellular functions of non-OSCC and OSCC in Figure 4.

### 4.6. Systematic Drug Discovery Based on the Drug/Target Interaction Prediction by the DNN-Based DTI Model and Drug Design Specifications for OSCC

Based on drug/target (biomarker) prediction and drug design specifications, we want to discover a potential multiple-molecule drug for the carcinogenic biomarkers on OSCC. First, we trained a DTI model based on a deep neural network (DNN) to predict the drug–target interactions between the drugs and targets (biomarkers). However, it is not enough to only consider the interactions between the molecular drugs and the targets in drug design. Some drug design specifications, i.e., adequate regulation ability, low toxicity and high sensitivity, are necessary to filter the candidate drugs predicted by the DNN-based DTI model. This systems drug discovery method is introduced in the following paragraphs for designing a multiple-molecule drug for OSCC treatment before clinical trials.

The flowchart of the systematic drug discovery method is shown in Figure 5. First, drug–target interaction databases by UniProt [84], DrugBank [59], ChEMBL [85] and Pubchem [86] are combined to train DNN as the DTI model for drug–target interaction prediction. In a few years, the feature-based method, i.e., molecular descriptor, will be widely used to describe the structural and chemical properties of molecules such as characteristics from the 2D, 3D spectrum of the structure, molecular weight, hydrophilic and hydrophobicity, etc. The chemical properties of the drug and genomic sequence of the target could be described with the molecular descriptor for the purpose of convenient analysis in drug design since the molecular descriptor can transform complicated chemical properties into a feature vector. We used the molecule descriptor function and protein descriptor function of python package pyBioMed to transform the drug and target into descriptors as drug and target features, respectively, under the python2.7 environment. Drug features of molecule descriptors include constitutional descriptors, connectivity indices, E-state indices, charge descriptors, molecular properties and kappa shape indices. For the target features, the protein descriptor calculates the structural and physicochemical features of proteins and peptides from the amino acid sequence such as amino acid composition, dipeptide composition, etc. For more detailed information about the descriptor transformation, readers can access the documents of pyBioMed. The drug descriptor and the target descriptor are combined into the feature vector corresponding to the drug–target pair, as follows:

(37) $$v_{\mathrm{drug}\text{–}\mathrm{target}}=[D,T]=[d_{1},d_{2},\cdots ,d_{M},t_{1},t_{2},\cdots ,t_{N}]$$

where the former symbol *D* in $v_{\mathrm{drug}\text{–}\mathrm{target}}$ is defined as the drug descriptor and the latter symbol *T* is the target descriptor. $d_{1}$ means the first drug feature; $t_{1}$ represents the first target feature; *M* indicates the total number of features of the drug and *N* expresses the total number of features of the target. We have 363 features for the drug and 996 features for the target.

Before training our DNN-based DTI model, we encountered several problems: features of different scales will affect our results—for example, large-scale features will have a great dominance in the training process. There are far more unverified data in the data than verified data, so there exists a between-class imbalance issue which could lead to a biased parameter updating tendency to the larger class during the training process.

To remedy the imbalance issue, we have to solve the problem of scale first. We use the min-max normalization to deal with this problem. Min-max normalization can handle this problem effectively, but compared to normalization, it is very sensitive to outliers. Additionally, feature normalization is performed before principal component analysis (PCA) to improve the DNN-based DTI model training performance. Therefore, the normalization to the features is given as follows:

(38) $$d_{i}^{*}=\frac{d_{i}-\mu_{i}}{\sigma_{i}},\;\forall i=1,\ldots ,M$$

(39) $$t_{j}^{*}=\frac{t_{j}-\mu_{j}}{\sigma_{j}},\;\forall j=1,\ldots ,N$$

where $d_{i}$ denotes the *i*-th drug feature and $d_{i}^{*}\;$ expresses the *i*-th drug feature after the standardization; $\mu_{i}$ and $\sigma_{i}$ respectively denote the mean and standard deviations of the *i*-th drug feature; $t_{j}$ means the *j*-th feature of the target and $t_{j}^{*}$ represents the *j*-th feature of the target after standardization; $\mu_{j}$ and $\sigma_{j}$ separately indicate the mean and standard deviation of the *j*-th target feature; *M* expresses the total number of drug features and *N* denotes the total number of target features.

In our data, the number of proven drug–target interaction samples, called the positive class, is 1455, and the number of unverified drug–target interaction samples, called the negative class, is much larger than the positive class. There exists a huge difference in the amount between the negative class and the positive class, which leads to the between-class imbalance issue. Before training the DNN-based DTI model, we also implemented data preprocessing by the principal component analysis (PCA) method—(35)–(40)—to reduce the dimension of the features of the drug and target to the dimension of the input of DNN. The PCA extraction was conducted after down-sampling and standardization to ensure that the PCA could accurately project the original data on the feature plane. It is worth noting that data preprocessing, e.g., PCA, is only conducted in training data because testing data should be deemed unknown to the model. The drug features were selected by the top 85% with higher singular values. The insignificant features of the drug and the target lower than the dimension of the input of DNN are deleted, and the remainders were employed to train DNN as the DTI model to predict candidate drugs for biomarkers (drug targets) of OSCC.

We referenced the basic concept and knowledge of DNN to train a DNN-based DTI model to predict drug–target interaction through the python Tensorflow and Keras package under the python3.7 environment. In the model structure of the DNN-based DTI model in Figure 4, the function in a feedforward step can be denoted as follows:

(40) h=σ(wx+b)

where x and h respectively denote the input and output; w  is the weighting matrix and b is the bias vector; σ (.)  indicates the activation function with ReLU in the hidden layer and the sigmoid function at the output layer. Since the binary classification issue is concerned, the binary-cross entropy is chosen as the cost function to calculate the model loss [11]:

(41) $$C_{n}(w,\;b)=-[{\hat{p}}_{n}\log p_{n}+(1-{\hat{p}}_{n})log(1-p_{n})]$$

(42) $$L(w,\;b)=\frac{1}{N}\sum_{n=1}^{N}C_{n}(w,\;b)$$

where $p_{n}$ means the truth label of positive interaction; ${\hat{p}}_{n}$ indicates the predictive probability of positive interaction, and $1-p_{n}$ shows the truth label of negative interaction; $1-{\hat{p}}_{n}$ represents the predicted probability of negative interaction; L(w, b) denotes the average of total loss $C_{n}\left(w,\;b\right)$. According to the cost function, the backward propagation algorithm is applied to update the model parameter set θ containing the weighting matrix *w* and the bias vector *b* through calculating the gradient of the cost function in (46) and eventually obtain the optimal solution $\theta^{*}$ in (48), as follows.

(43) $$\theta =\left[\begin{array}{c} w\\ b \end{array}\right]$$

(44) $$\theta^{*}=\underset{\theta }{\mathrm{argmin}}L(\theta )$$

(45) $$\theta^{l}=\theta^{l-1}-\eta \nabla L(\theta^{l-1})$$

where l is the l-th epoch of the learning procedure; η is learning rate and $\nabla L\left(\theta^{l-1}\right)$ is the gradient of $L\left(\theta^{l-1}\right),$ as follows:

(46) $$\nabla L(\theta^{l-1})=\left[\begin{array}{c} \frac{\partial L(\theta^{l-1})}{\partial w}\\ \frac{\partial L(\theta^{l-1})}{\partial b} \end{array}\right]$$

Based on the backward propagation method, the DNN-based DTI model could adjust the parameters to fit the drug–target interaction data at each iteration well.

In addition, the hyperparameters were tuned to not only lower the training time but also achieve the best model performance. We used an optimizer with default settings and set the learning rate to 0.003 to make model parameter θ converge faster and precisely. We set 100 for epochs and 100 for batch size. For the data, we split one-fourth of the data as testing data and three-fourths of it as training data. Moreover, we divided the training data into five equal folds to perform the five-fold cross-validation strategy. In the five-fold data, four-fifths of them were for the model training and one-fifth of them was used as the validation data, which play the role of supervisor to check whether the model was better than that of the former epoch. Additionally, five-fold cross-validation could verify the stability of the data and model. To avoid model overfitting, we applied an early stopping strategy to check if the test accuracy decreased when the training accuracy increased continuously. Moreover, we embedded the dropout layer after each hidden layer to further prevent model overfitting and set 0.4 for the dropout rate. After training the DNN-based DTI model, we adopted a performance measurement AUC (area under the curve) score and ROC (receiver operating characteristics) curve in Figure 8 to check the model performance. It is one of the most useful evaluation metrics to visualize the model performance when it comes to the classification problems. The higher the AUC score (which means the area under the line is larger), the better the accuracy is for the DNN-based DTI model in predicting the true positive and true negative drug–target interaction. The formulas for the AUC score and ROC curve are shown below [83].

(47) $$\mathrm{TPR}(\mathrm{True}\;\mathrm{Positive}\;\mathrm{Rate})=\frac{\mathrm{TP}}{\mathrm{TP}+\mathrm{FN}}$$

(48) $$\mathrm{specificity}=\frac{\mathrm{TN}}{\mathrm{TN}+\mathrm{FP}}$$

(49) $$\mathrm{FPR}(\mathrm{False}\;\mathrm{Positive}\;\mathrm{Rate})=1-\mathrm{specificity}=\frac{\mathrm{FP}}{\mathrm{TN}+\mathrm{FP}}$$

where TP (True Positive) means that the real value is true and is judged correctly; TN shows that the real value is true and is judged by mistake; FP indicates that the real value is false and is judged accurately; FN represents that the real value is false and is judged in error.

## 5. Conclusions

In this study, based on our proposed combination of systems biology and systems drug discovery design methods, we investigated the complex carcinogenic molecular mechanisms of OSCC from genome-wide data, genetic and epigenetic network perspectives, and designed prospective multiple-molecular drugs as a multi-molecule drug to target multiple biomarkers of OSCC. We construct a genetic and epigenetic biological network by exploring methods of systematic identification and systematic sequential detection of big data. Afterwards, we extracted core signaling pathways by the PNP method and KEGG pathway annotation to select important biomarkers from OSCC carcinogenesis by comparing core signaling pathways and their downstream abnormal cellular functions. To discover drug candidates that interact with these biomarkers, we trained a DNN-based DTI model by DTI databases to predict drug–target interaction probability values. In addition, we took the drug regulation ability, low toxicity and high sensitivity as the drug design criteria to screen out suitable potential drugs from the predicted drug candidates. Therefore, a set of combined multi-molecular drugs is proposed as a multi-molecule drug for OSCC treatment. In the future, more and more different types of genomics data will be available for epigenetic and epigenetic regulations. A combination of multiple types of genomics data is needed to help us enhance our work and gain a deeper understanding of the significant biomarkers of the carcinogenic mechanism of OSCC. It is anticipated that the proposed systems biology and systems drug discovery design approach may provide new directions for OSCC treatment.

## Figures and Tables

**Table 1:** Samples of microarray data from GSE30784 and GSE17913.

Microarray Data	Non-OSCC	OSCC
GSE30784 and GSE17913	102	167

**Table 2:** The statistics of the nodes in real GWGEN and core GWGEN of OSCC.

	Real GWGEN of OSCC	Core GWGEN of OSCC
Protein	13,855	4621
Receptor	2112	672
TF	1492	620
miRNA	31	2
LncRNA	419	85
Total nodes	17,909	6000

**Table 3:** KEGG pathway enrichment analysis of core OSCC signaling pathways.

KEGG Pathway Enrichment Analysis of OSCC Core Signaling Pathways		
Pathway	Gene number	*p*-value
Cell cycle	91	8.3 × 10^−20^
Pathways in cancer	267	3.1 × 10^−19^
MAPK signaling pathway	149	3.6 × 10^−11^
Apoptosis	80	3.2 × 10^−10^
Proteoglycans in cancer	120	1.0 × 10^−14^

**Table 4:** KEGG pathway enrichment analysis of core non-OSCC signaling pathways.

KEGG Pathway Enrichment Analysis of Non-OSCC Core Signaling Pathways		
Pathway	Gene number	*p*-value
Cellular senescence	95	2.3 × 10^−13^
MAPK signaling pathway	166	2.4 × 10^−18^
Human T-cell leukemia virus 1 infection	134	3.2 × 10^−18^
Cell cycle	84	4.2 × 10^−15^
P53 signaling pathway	46	1.6 × 10^−7^

**Table 5:** Some candidate drugs for biomarkers of OSCC and their regulability, toxicity and sensitivity information.

HES1 (-)			
Drug	Regulation Ability (L1000)	Sensitivity (PRISM)	Toxicity (LC50, mol/kg)
capsaicin	0.1690	−0.1217	4.202
gabazine	0.3716	−0.6103	3.079
phenolphthalein	−0.6116	−0.4833	5.297
tetramisole	0.1036	0.1136	4.111
gefitinib	0.2750	−0.5144	5.068
**TCF (-)**			
Drug	Regulation Ability (L1000)	Sensitivity (PRISM)	Toxicity (LC50, mol/kg)
carvedilol	−0.0787	−0.0906	5.014
fipronil	−0.1207	−0.0939	5.534
metformin	0.0770	−0.0789	2.039
diethylcarbamazine	0.0501	−0.0848	2.008
dyphylline	0.1372	0.0356	2.022
**NF-κB (+)**			
Drug	Regulation Ability (L1000)	Sensitivity (PRISM)	Toxicity (LC50, mol/kg)
sirolimus	−0.0866	−0.2058	3.486
terfenadine	−0.7665	−0.7406	5.437
metformin	−0.2607	−0.0789	2.039
gallic-acid	−1.0620	0.6208	3.262
gefitinib	−0.3428	−0.5144	5.068
**SP1 (+)**			
Drug	Regulation Ability (L1000)	Sensitivity (PRISM)	Toxicity (LC50, mol/kg)
niridazole	−0.6456	−0.1400	2.746
chlorambucil	−0.0559	−0.1424	3.249
bepridil	0.7249	0.2789	5.083
gallic-acid	−0.5239	0.6208	3.262
disopyramide	−0.3694	−0.1440	3.316

(+), abnormal overexpression; (-), abnormal low expression.

**Table 6:** A potential multiple-molecule drug for OSCC from the candidate molecular drugs in Table 5 by the drug design specifications of suitable regulation ability, low toxicity and high sensitivity.

	Targets	HES1	TCF	NF-κB	SP1	Toxicity (LC50, mol/kg)	Sensitivity (PRISM)
Drugs							
metformin			✓(0.0770)	✓(−0.2607)		2.039	−0.0789
gefitinib		✓(0.2750)		✓(−0.3428)		5.068	−0.5144
gallic-acid				✓(−1.0620)	✓(−0.5239)	3.262	0.6208
metformin				gefitinib			
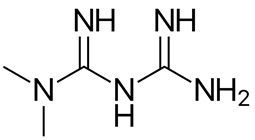				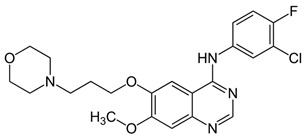			
gallic-acid							
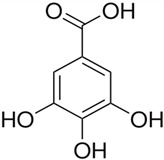							

✓ denotes the drug that can bind to the biomarker with a desired regulation ability in (‧).

## Data Availability

The gene raw count datasets of human genes are integrated from GSE30784 (https://www.ncbi.nlm.nih.gov/geo/query/acc.cgi?acc=GSE30784, accessed on 1 November 2021) and GSE17913 (https://www.ncbi.nlm.nih.gov/geo/query/acc.cgi?acc=GSE17913, accessed on 1 November 2021). The drug regulation ability data are from Phase I L1000 Level 5 datasets (https://www.ncbi.nlm.nih.gov/geo/query/acc.cgi?acc=GSE92742, accessed on 1 November 2021). The drug sensitivity datasets are from DepMapPRISM primary screen datasets (https://depmap.org/repurposing/, accessed on 1 November 2021).
